# *N*-*tert*-Butanesulfinyl imines in the asymmetric synthesis of nitrogen-containing heterocycles

**DOI:** 10.3762/bjoc.17.86

**Published:** 2021-05-12

**Authors:** Joseane A Mendes, Paulo R R Costa, Miguel Yus, Francisco Foubelo, Camilla D Buarque

**Affiliations:** 1 Department of Chemistry, Pontifical Catholic University of Rio de Janeiro Puc-Rio, CEP 22435-900, Brazil; 2 Laboratory of Bioorganic Chemistry, Institute of Research of Natural Products, Health Science Center, Federal University of Rio de Janeiro UFRJ, CEP 21941-590, Brazil; 3 Centro de Innovación en Química Avanzada (ORFEO-CINQA), Universidad de Alicante, Apdo.99, 03080 Alicante, Spain; 4 Departamento de Química Orgánica, Facultad de Ciencias, Universidad de Alicante, Apdo. 99,03080 Alicante, Spain; 5 Instituto de Síntesis Orgánica (ISO), Universidad de Alicante, Apdo. 99, 03080 Alicante, Spain

**Keywords:** asymmetric synthesis, chiral auxiliary, natural products, nitrogen-containing heterocycles, *N*-*tert*-butanesulfinyl imines

## Abstract

The synthesis of nitrogen-containing heterocycles, including natural alkaloids and other compounds presenting different types of biological activities have proved to be successful employing chiral sulfinyl imines derived from *tert*-butanesulfinamide. These imines are versatile chiral auxiliaries and have been extensively used as eletrophiles in a wide range of reactions. The electron-withdrawing sulfinyl group facilitates the nucleophilic addition of organometallic compounds to the iminic carbon with high diastereoisomeric excess and the free amines obtained after an easy removal of the *tert*-butanesulfinyl group can be transformed into enantioenriched nitrogen-containing heterocycles. The goal of this review is to the highlight enantioselective syntheses of heterocycles involving the use of chiral *N*-*tert*-butanesulfinyl imines as reaction intermediates, including the synthesis of several natural products. The synthesis of nitrogen-containing heterocycles in which the nitrogen atom is not provided by the chiral imine will not be considered in this review. The sections are organized according to the size of the heterocycles. The present work will comprehensively cover the most pertinent contributions to this research area from 2012 to 2020. We regret in advance that some contributions are excluded in order to maintain a concise format.

## Intoduction

**Scheme 1:**
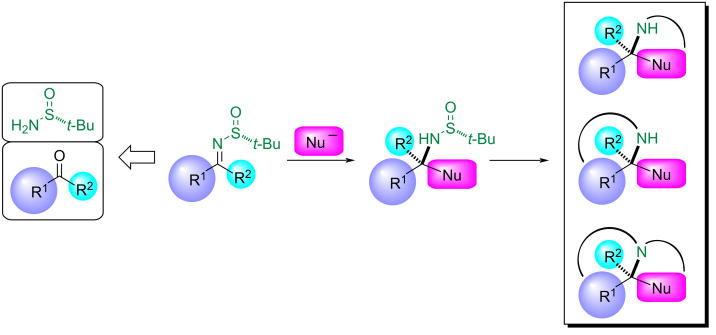
General strategy for the enantioselective synthesis of *N*-containing heterocycles from *N*-tert-butanesulfinyl imines.

Chiral imines derived from *tert*-butanesulfinamide have been extensively used as electrophiles in a wide range of reactions. The presence of the chiral electron-withdrawing sulfinyl group facilitates the nucleophilic addition of organometallic compounds to the iminic carbon [1–3]. The ready availability of both enantiomers of *tert*-butanesulfinamide in large-scale processes, the easy deprotection of the amine under mild acidic conditions, and a practical procedure for recycling the chiral auxiliary [4–5] have contributed to the widespread use of these imines as precursors of chiral compounds with a nitrogen atom bonded to a stereogenic center. The amine derivatives, resulting after removal of the *tert*-butanesulfinyl group, can be transformed into enantioenriched nitrogen-containing heterocycles [6–7] including natural alkaloids [8–11] and other compounds that show different types of biological activities [12–13]. The way to achieve these transformations is by intramolecular cyclizations, involving the free primary amine, and appropriate reactive positions (those positions bearing a leaving group) in the electrophile or in the carbonyl component of the starting imine (Scheme 1).

### Synthesis of *tert*-butane *N*-sulfinyl imines

**Scheme 2:**
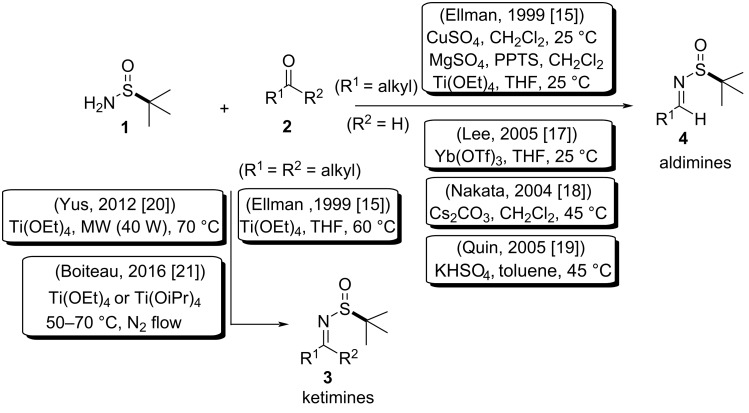
Methodologies for condensation of aldehydes and ketones with *tert*-butanesulfinamides (**1**).

The first method developed for the synthesis of enantiomerically pure *N*-*tert*-butanesulfonamide **1** was reported by Ellman and co-workers [14–15]. In 1999, they described the synthesis of imines from the condensation reaction of aldehydes or ketones with *tert*-butanesulfinamides. In this work, the condensation with aldehydes was carried using Ti(OEt)_4_ in tetrahydrofuran (THF), or CuSO_4_ in dichloromethane at room temperature. The combination of MgSO_4_ in the presence of a catalytic amount of pyridinium *p*-toluenesulfonate (PPTS) also worked well to perform these condensations [1,16]. For the formation of aldimines, other methodologies are described in the literature using condensation reagents such as Yb(OTf)_3_ [17], Cs_2_CO_3_ [18] and KHSO_4_ [19]. However, for the synthesis of ketimines, Ti(OEt)_4_ was the only effective reagent when performing the reaction at 60 °C in THF [16]. Ketimines were also synthesized with Ti(OEt)_4_, under microwave irradiation in a solvent-free system [20]. In hindered ketones, Ti(OiPr)_4_ or Ti(OEt)_4_ using vacuum or under a nitrogen flow were effective to *tert*-butanesulfinyl ketimine condensation (Scheme 2) [21].

### Mechanism of addition of nucleophiles to *N*-sulfinyl imines

**Scheme 3:**
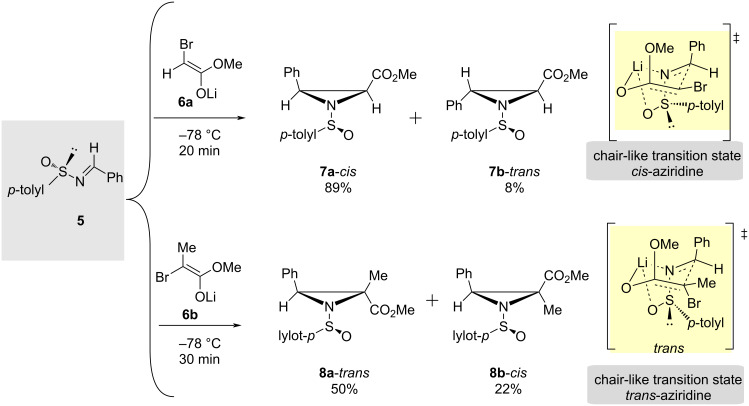
Transition models for *cis*-aziridines and *trans*-aziridines.

The *p*-toluenesulfinamide **5** was first described by Davis and co-workers in a racemic form [22], and subsequently, the compound was prepared and isolated as a single enantiomer [23–24], becoming an important tool in the asymmetric synthesis of aziridines [25–26], α-amino acids [27–28], β-amino acids [23,29] and branched α-amines [30–31]. The Darzens-type asymmetric synthesis of *N*-(*p*-toluenesulfinyl)aziridine 2-carboxylate esters (**7** and **8**) was described through the addition of lithium α-bromoenolates to enantiopure *p*-toluenesulfinamide **5**. *cis*-aziridine **7a** was formed as the major diastereoisomer in 89% yield and the *trans-*isomer in 8% yield in a one-step procedure using lithium enolates of methyl bromoacetate **6a** and sulfinyl imine **5.** Lithium enolates of methyl α-bromopropionate gave *trans*-aziridine in 50% yield under the same conditions. The transition state is proposed with a six-membered chair-like transition containing a four-membered metallocycle. In *cis*-aziridine the enolate of methyl α-bromoacetate has *E*-geometry and the *trans*-aziridine **8a** has *Z*-geometry [32–33]. In the transition state, the metal cation of the enolate is being coordinated with both nitrogen and oxygen atoms of the sulfinimine [25–26] (Scheme 3).

**Scheme 4:**
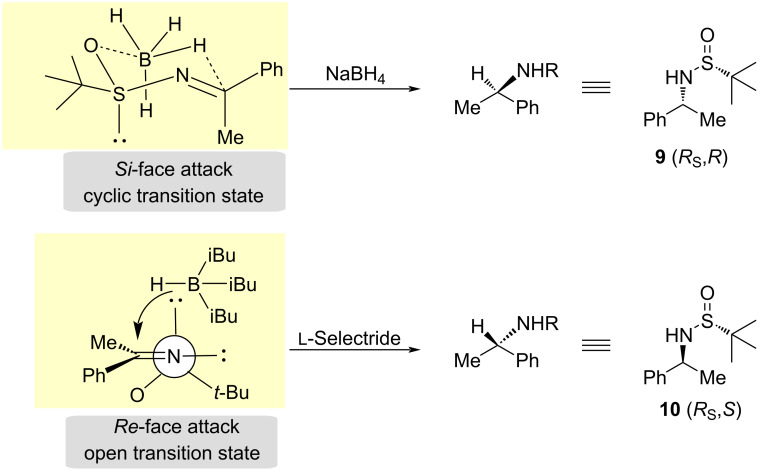
Mechanism for the reduction of *N-tert*-butanesulfinyl imines.

In 1999, Ellman and co-workers described the reduction of sulfinyl imines using sodium borohydride (NaBH_4_) [34] or o ʟ-selectride [35]. Davis–Ellman transition state models were proposed to rationalize organometallic additions to *N*-sulfinyl imines. The mechanism for obtaining these two stereoisomers was elucidated in the work published by Andersen and co- workers [36]. The origin of the reversal of the diastereofacial selectivity on the change of reducing agents is based on the operating transition states [37–38]. A cyclic transition state is proposed in the reaction with sodium borohydride. In this transition state, the oxygen of the sulfinyl group interacts with the boron atom, facilitating the release of the hydride, directing the attack to the *Si*-face of the imine with (*R*_S_,*E*) configuration. When the reduction is performed with ʟ-selectride, with the poorly coordinating metal hydride, an open transition state operates due to the bulkiness of the isobutyl groups bonded to the boron atom. In this case, the attack of the hydride takes place to the less hindered *Re*-face of the imine (Scheme 4) [1,36].

The nucleophilic addition reactions to *N-tert*-butanesulfinyl imines were also described by Ellman and co-workers who reported the addition of allylmagnesium bromide to ketimines. The employment of Grignard reagents showed greater diastereoselectivity than reactions using organolithium and organocerium compounds. In some examples, the use of organolithium is feasible through the use of aluminum-derived additives [15]. In this study, the influence of solvents on diastereoselectivity was also observed. They found that the reactions performed in noncoordinating solvents, such as toluene and dichloromethane, took place with high diastereoselectivity. However, solvents such as ether and THF had a negative impact on stereoselectivity [15]. On the other hand, recent studies developed by Sirvent and Foubelo demonstrated the influence of the solvents in both the yield and diastereoselectivity in these reactions. They found that working in THF led to higher yields and poorer diastereoselectivities than when the reactions were performed in less coordinating solvents, such as diethyl ether and toluene [39].

**Scheme 5:**
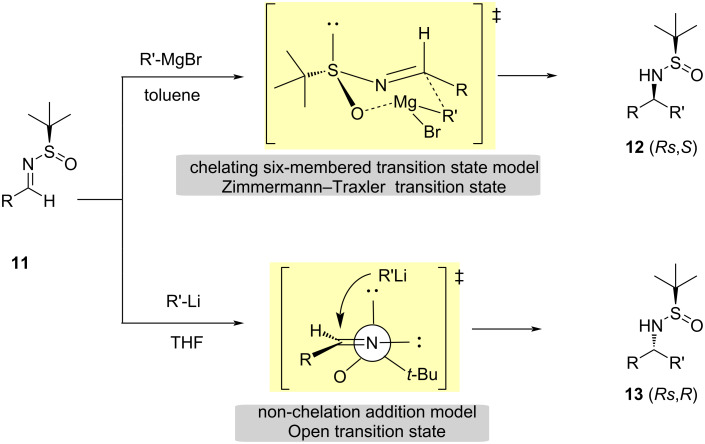
Transition models for the addition of organomagnesium and organolithium compounds to *N*-tert-butanesulfinyl imines.

Based on a broader analysis related to the effects of the solvent, metal and additives in 1,2-addition reactions to *N-tert*-butanesulfinyl imines of organometallic compounds, different transition models have been proposed to explain the stereochemical outcomes. The cyclic model justified by the Zimmermann–Traxler transition state [40–42] is the typical mechanism operating in reactions involving Grignard reagents in noncoordinating solvents, such as toluene and dichloromethane, while an acyclic model [43] is common in organolithium compounds in solvents such as THF. In the cyclic model, the bulky *tert*-butyl group occupies an equatorial position due to steric hindrance [1,14,44–45] (Scheme 5).

Other contributions to the nucleophilic addition reactions to *N-tert*-butanesulfinyl imines were made by Yus and co-workers employing organozincates [46–49] and indium [50].

After this brief about the synthesis of enantiomerically pure *N*-*tert*-butanesulfonamide and applications in some nucleophilic additions, the next sections will describe the synthesis of several alkaloids according to the size of the heterocycles. We regret in advance that some contributions are excluded in order to maintain a concise format.

## Review

### Asymmetric synthesis of aziridines

Saturated nitrogen-containing three-membered heterocycles have attracted increasing interest in recent years because compounds with this structural motif display quite diverse pharmacological activities. Chiral aziridines [51] also play an important role in asymmetric synthesis because they can act both as ligands [52–55] and as chiral auxiliaries [56]. The most widely used synthetic methods to form the aziridine ring [57–61] include intramolecular cyclizations in amines bearing potential leaving groups. Stereoselective syntheses of aziridines have been successfully carried out by combining a nucleophilic addition to *N*-*tert*-butanesulfinyl α-chloroimines, and an intramolecular cyclization, the chlorine atom being finally displaced. Chiral *N*-*tert*-butanesulfinyl aldimines and ketimines have also been used successfully to form aziridines through aza-Darzens and Corey–Chaykovsky reactions [62–63].

**Scheme 6:**
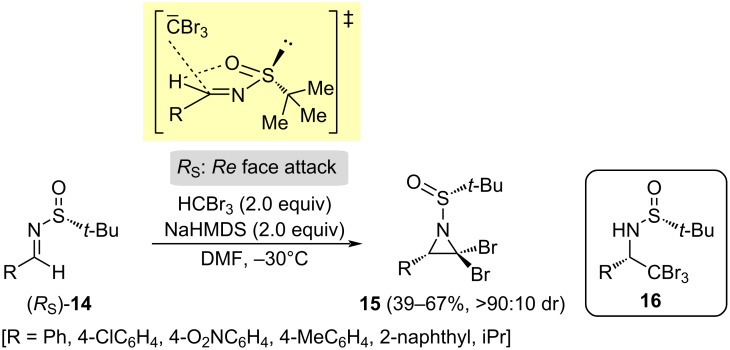
Synthesis of 2,2-dibromoaziridines **15** from aldimines **14** and bromoform, and proposed non-chelation-controlled transition state model.

The first asymmetric synthesis of 2,2-dibromoaziridines **15** was achieved by performing the nucleophilic addition of the anion resulting from the deprotonation of bromoform with sodium hexamethyldisilazide (NaHMDS) to chiral *N-tert*-butanesulfinyl aldimines (*RS*)-**14**, at low temperature, and using DMF as solvent. After addition, a subsequent intramolecular cyclization involving the resulting amide and the vicinal carbon with bromine atoms took place. By contrary, when the reaction was carried out in THF, the elimination process was suppressed, leading exclusively to enantiomerically pure α-tribromomethylamines **16**. The structure and configuration of aziridines **15** were determined unambiguously by single crystal X-ray analysis [64]. In order to explain the experimental results, a nonchelation controlled transition state was proposed. Based on computational studies, it is known that a kind of s-*cis* arrangement of the sulfinyl group is the most stable conformation of the imine, due to the contribution of the hydrogen bonding of the oxygen and the iminic hydrogen. In this scenario, the tribromomethyl anion attacked the less hindered *Re* face of the imine with (*RS*) configuration (Scheme 6).

**Scheme 7:**
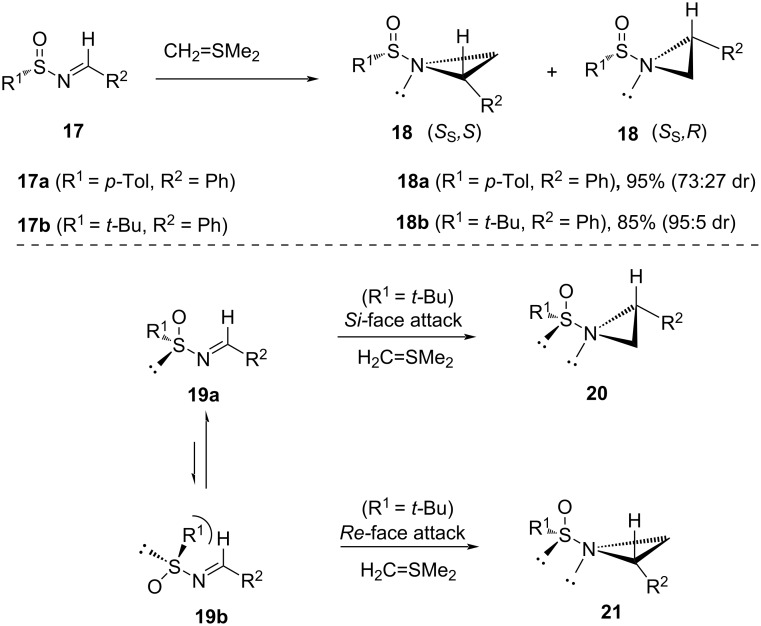
Diastereoselective synthesis of aziridines from *tert*-butanesulfinyl imines.

The applicability of sulfinyl imines was also shown by Garcia Ruano and co-workers. (*S_S_*)-*tert*-Butanesulfinyl imine **17b** provided better diastereoselectivity to obtain aziridines **18b** than (*S_S_*)-*tert*-butanesulfinyl imine **17a** to obtain aziridines **18a** (Scheme 7) [65].

**Scheme 8:**
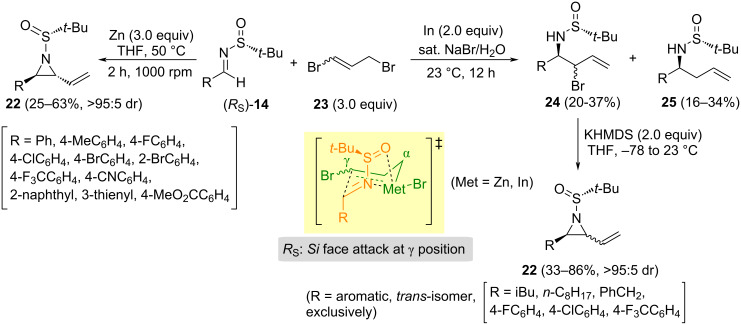
Synthesis of vinylaziridines **22** from aldimines **14** and 1,3-dibromopropene **23**, and proposed chelation controlled transition state model.

Allylation of *N*-*tert*-butanesulfinyl imines **14** with allylic bromides in the presence of zinc or indium metals is a well-known reaction [66–67]. It is possible to control and predict the stereochemistry of the addition to get the corresponding homoallylamine derivative with a high level of stereocontrol. The reaction of chiral imine **14** with an excess of 1,3-dibromopropene (**23**) in THF at 50 °C for 2 h, and a 1000 rpm rotation speed, led to *trans*-vinylaziridines **22**, in moderate yields and high diastereoselectivities. Those were the reaction conditions that Sun and co-workers found to be optimal for the formation of the vinylaziridines [68]. However, when the allylation was performed in the presence of indium metal, in a saturated aqueous solution of sodium bromide, a mixture of the bromoallylation and allylation products **25** and **26**, respectively, were obtained [69]. The addition of the allyl unit to the imines proceeded with total facial diastereoselectivity, producing also preferably diastereoisomers with *anti* relative configuration. According to the mechanism depicted on Scheme 8, the allyl unit reacted at γ-position, taking place the addition to the *Si* face of the imines with *R*_S_ configuration. The bromoallylated product **24** was obtained as a mixture of *anti*/*syn* diastereoisomers. Treatment of compounds **24** with potassium hexamethyldisilazide provided vinylaziridines **22** through an intramolecular cyclization step. This intramolecular nucleophilic substitution is a stereospecific process. In the case of aromatic compounds **24**, *trans*-vinylaziridines were the only reaction products, meanwhile for aliphatic derivatives **24** (R = alkyl), *trans-* and *cis*-aziridines **22** were isolated in practically the same ratio as the *anti*/*syn* ratio of their precursors **24**. This shows that the cyclization reaction is stereospecific (Scheme 8). Comparing both methodologies, the indium-mediated bromoallylation seemed to be superior, since aliphatic aldimines **14** were compatible with this approach.

**Scheme 9:**
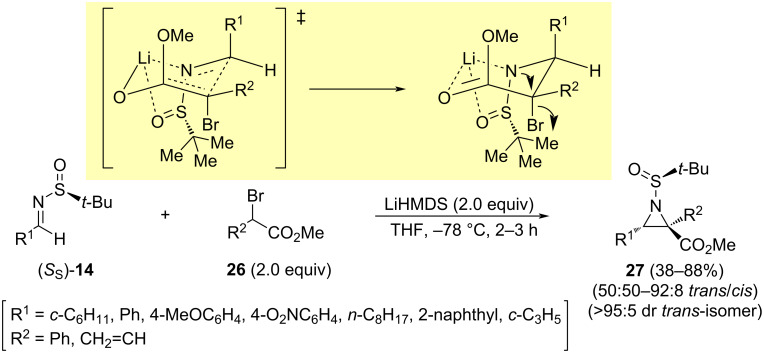
Synthesis of vinylaziridines **27** from aldimines **14** and α-bromoesters **26**, and proposed transition state leading to the major diastereoisomer.

The group of Stockman reported the synthesis of 2,2′,3-substituted aziridines **27** from *N*-*tert*-butanesulfinyl imines **14** and α-bromoesters **26** by applying an aza-Darzens methodology [70]. The reactions were performed in THF at −78 °C, using lithium hexamethyldisilazide as base. Aziridines with relative *trans*-configuration were obtained in good yields and excellent stereoselectivities with methyl α-bromo-α-phenylacetate (**26**, R^2^ = Ph). Lower yields, and poorer diastereoselectivities were observed with less bulky methyl 2-bromo-2-butenoate (R^2^ = CH_3_CH=), leading to an almost complete loss of *cis*/*trans* selectivity by the reaction with aliphatic aldimines **14**. The absolute configuration of the reaction products was unambiguously determined after X-ray crystallographic analysis of some of the reaction products. In order to rationalize the observed stereochemical outcome, a six-membered cyclic transition state has been proposed. The addition to imine **14** with *S*_S_ configuration takes place on the less hindered *Re* face, on the other hand, the *cis*/*trans* selectivity observed being a consequence of the *E* stereochemistry of both the imine **14** and the enolate derived from bromoester **26** (Scheme 9).

**Scheme 10:**
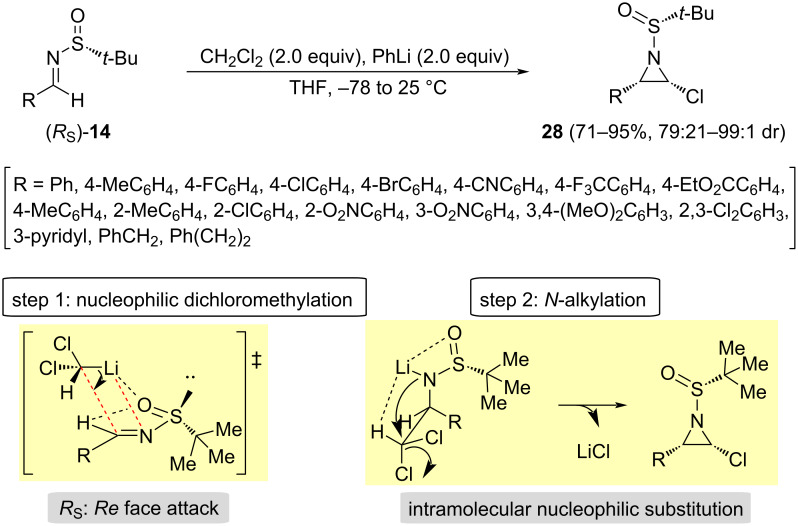
Synthesis of 2-chloroaziridines **28** from aldimines **14** and dichloromethane, and proposed transition state model for the nucleophilic addition and for the elimination step.

A two-step protocol carried out in a single synthetic operation was developed by Chen and Zhang to synthesize 3-substituted 2-chloroaziridines with relative *cis* configuration [71]. The reaction of chiral imines **14** in dichloromethane in the presence of 2 equivalents of phenyllithium at −78 °C to room temperature produced the expected 2-chloroaziridines with excellent yields and diastereoselectivities. Under the previously commented optimized reaction conditions, dichloromethyllithium is first generated, taking place a diastereoselective nucleophilic dichloromethylation of the imine. The addition took place almost exclusively to the *Re* face of the imine (*R*_S_)-**14**, which is the less sterically hindered in the most stable s-*cis* conformation (see Scheme 2). The second intramolecular *N*-alkylation step produced the 2-chloroaziridines **28** with relative *cis* configuration. The absolute proposed configurations were confirmed by X-ray crystallographic analysis (Scheme 10).

**Scheme 11:**
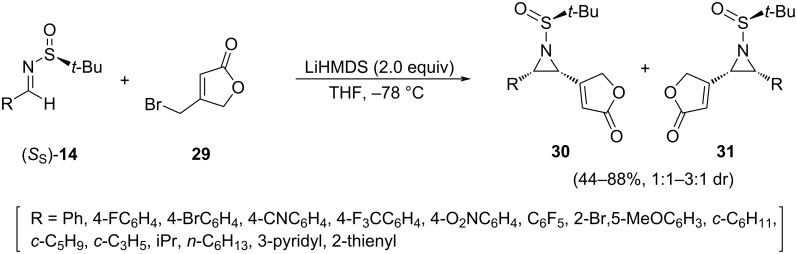
Synthesis of *cis*-vinylaziridines **30** and **31** from aldimines **14** and bromomethylbutenolide **29***.*

An interesting asymmetric vinylogous aza-Darzens reaction was employed to access *cis*-vinylaziridines **30** and **31**. The group of Njardarson found that the reaction of different aromatic and aliphatic chiral imines (*S*_S_)-**14** with the dienolate resulting from the deprotonation of bromomethyl butenolide **29** in THF at −78 °C led to a mixture of diastereomeric *cis*-vinylaziridines **30** and **31** with good yields in most cases. Lithium hexamethyldisilazide was the base of choice to perform the deprotonation, and it must be added very slowly to the reaction mixture in order to suppress self-dimerization of the butenolide [72]. The structures as well as the absolute and relative stereochemistry of reaction products **30** and **31** were also unambiguously determined following a single-crystal X-ray analysis (Scheme 11).

**Scheme 12:**
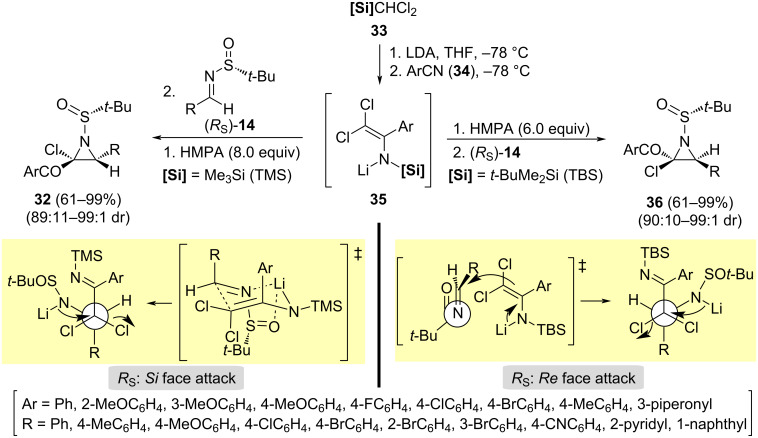
Synthesis of 2-chloro-2-aroylaziridines **36** and **32** from aldimines **14**, arylnitriles **34**, and silyldichloromethanes **33**, and proposed transition states.

The stereoselective synthesis of diastereomeric 2-chloro-2-aroylaziridines **36** and **32** was successfully accomplished through a three-component cascade coupling reaction of silyldichloromethanes **33**, arylnitriles **34** and chiral *N*-*tert*-butanesulfinyl aldimines (*R*_S_)-**14**. The process reported by Lu, Xu and co-workers started with the deprotonation of silyl compounds **33** with LDA at –78 °C, leading to the corresponding silyldichloromethyllium derivative, which reacted with arylnitrile **34**. After nucleophilic addition and [1,3]-aza-Brook rearrangement, *N*-silyllithiumenamide **35** was formed. This strongly nucleophilic species could be traped by the chiral imine (*R*_S_)-**14**, producing 2-chloro-2-aroylaziridines via and aza-Darzens reaction [73]. Importantly, the structure of the final aziridine is determined by the silyl group, and the order of the addition of HMPA and imine **14** in the multicomponent coupling. When the bulky TBS group was used, and HMPA was added to the reaction mixture before the imine **14**, aziridines **36** were formed. The addition of lithium metaloenamine took place through and open transition state to the *Re* face of the imine (*R*_S_)-**14**, followed by an intramolecular nucleophilic substitution to form the aziridines ring. On the contrary, aziridines **32** were obtained starting from dichloromethyltrimethylsilane (**33**, [Si] = TMS), and adding the chiral imine before HMPA to the reaction mixture. The nucleophilic addition of metaloenaime occurred through a cyclic transition state to the *Si* face of the imine (*R*_S_)-**14**. In this way, both *cis-*aziridines diastereoisomers **36** and **32** were formed from the same chiral imine **14** and arylnitriles **34** (Scheme 12).

**Scheme 13:**
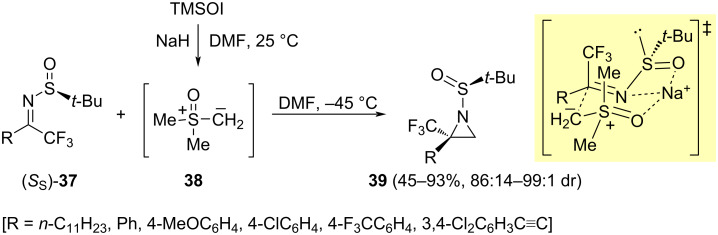
Synthesis of trifluoromethylaziridines **39** and proposed transition state of the aziridination.

Chiral sulfinyl imines have been also used in the stereoselective synthesis of aziridines. The reaction of *N*-*tert*-butanesulfinyl trifluoromethyl ketimines (*S*_S_)-**37** with dimethylsulfoxonium methylide **38** gave trifluoromethylated aziridines **39** in moderate to excellent yields (45–93%), and good diastereoselectivities (86:14 to >99:1 dr). The absolute configuration of compounds **39** was determined by X-ray crystallographic analysis, and it was found that the configuration of the newly generated stereocenter was *R*. Huang and co-workers proposed a cyclic transition state determined by the coordination of the nitrogen of the imine, and the oxygen atoms of the sulfoxonium and sulfinyl units to the sodium cation, which is present in the reaction medium [74]. The trifluoromethyl group occupies an equatorial position to avoid electrostatic repulsion with the lone pair of electrons of the sulfinyl group. The nucleophilic attack took place to the *Si* face of the ketimine **37** (Scheme 13).

**Scheme 14:**
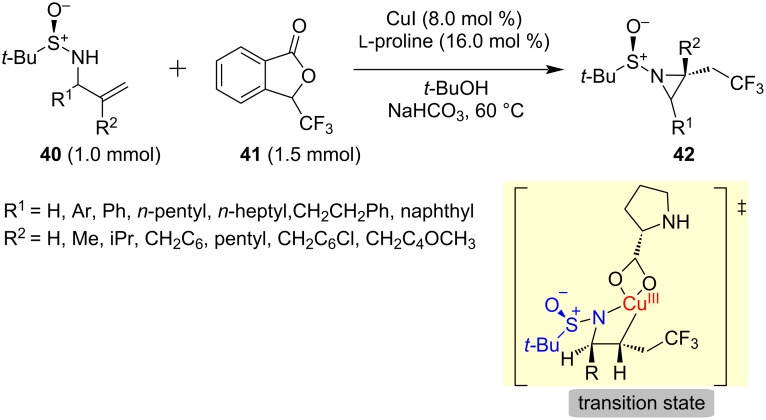
Synthesis of aziridines **42** and proposed state transition.

Recently, Yang and co-workers described the diastereoselective synthesis of aziridines **42** in one-step using the Cu(I)/ʟ-proline complex as a catalyst and *N*-*tert*-butasulfinylamide in an aminotrifluoromethylation reaction of alkenes. All the aziridines **42** were obtained with high diastereoselectivity (dr > 25:1) and good yields (56–98%) from allylic sulfonamides **40** and Togni’s reagent (**41**). The reaction mechanism is proposed based on DFT calculations. In this study, they observed an intramolecularly intermediate Cu(III) species, and the sulfinamide acts as a direction group and nucleophile [75] (Scheme 14).

### Asymmetric synthesis of azetidines

Azetidine [76–77] has attracted less attention than aziridines, pyrrolidines and piperidines, among small and medium size aza-heterocycles, because there are no general methods for their preparation. However, four-membered nitrogen-containing heterocycles have recently found applicability in pharmacy as highly biological active compounds. Among this group of nitrogenated heterocycles, β-lactams (azetidin-2-ones) have reached especial attention [78–80], being easily accessible from β-aminoesters.

**Scheme 15:**
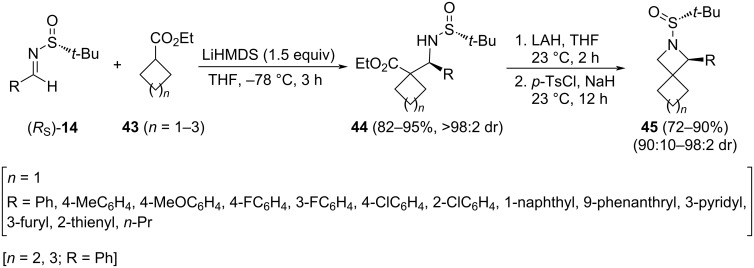
Synthesis of 1-substituted 2-azaspiro[3.3]heptanes, 1-phenyl-2-azaspiro[3.4]octane and 1-phenyl-2-azaspiro[3.5]nonane **45** from chiral imines **14**, and ethyl cycloalkanecarboxylates **43**.

A stereoselective synthesis of 1-substituted 2-azaspiro[3.3]heptanes **45** (*n* = 1) was reported by the group of Reddy [81] starting from ethyl cyclobutanecarboxylate **43** and chiral *N*-*tert*-butanesulfinyl aldimines (*R_S_*)-**14**. In this three-step procedure, a highly diastereoselective addition of the ethyl cyclobutanecarboxylate anion occurred first, followed by the reduction of the ester group, and an intramolecular nucleophilic substitution of the tosylate of the resulting primary alcohol (Scheme 15). This methodology was applicable to the synthesis of 1-phenyl-2-azaspiro[3.4]octane (**45**, *n* = 2, R = Ph) and 1-phenyl-2-azaspiro[3.5]nonane (**45**, *n* = 3, R = Ph). The structure and absolute stereochemistry of these compounds were assigned based on the single-crystal X-ray diffraction analysis of azaspiroheptane **45** with a 9-fenanthryl substituent at 1-position.

**Scheme 16:**
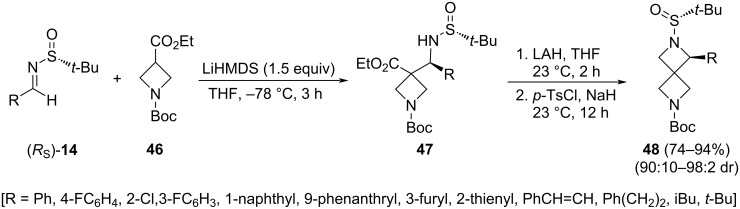
Synthesis of 1-substituted 2,6-diazaspiro[3.3]heptanes **48** from chiral imines **14** and 1-Boc-azetidine-3-carboxylate (**46**).

The same three-step procedure was applied by Reddy and co-workers to synthesize 1-substituted 2,6-diazaspiro[3.3]heptanes **48**, starting in this case from 1-Boc-azetidine-3-carboxylate **46**, instead of ethyl cyclobutanecarboxylate **43** [82]. This structural motif was found to have similar physicochemical properties as 2-substituted piperazines, which are key intermediates in drug discovery. The applied protocol was found to be practical for the asymmetric synthesis of a variety of aromatic, heteroaromatic, and aliphatic 1-substituted 2,6-diazaspiro[3.3]heptanes **48**, with overall yields ranging from 74 to 94% (Scheme 16).

### Asymmetric synthesis of β–lactams

**Scheme 17:**
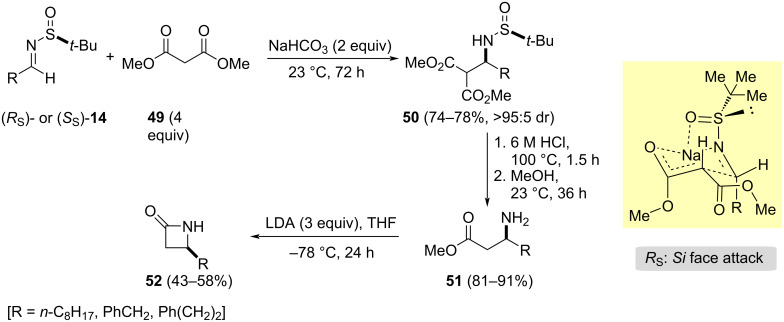
Synthesis of β-lactams **52** from chiral imines **14** and dimethyl malonate (**49**).

β-Lactam antibiotics are important drug class of antibacterial agents [83] and in the literature, several strategies are reported using the sulfinyl group [84–88]. In this context, an enantioselective synthesis of 4-substituted azetidin-2-ones **52** was also accomplished starting from chiral imines **14** and dimethyl malonate (**49**). A diastereoselective coupling of these components under solvent-free conditions was carried out, using sodium carbonate as base promoter. The resulting dimethyl 2-(1-aminoalkyl)malonates **50** were obtained in moderate to good yields as single diastereoisomers in all cases except for aromatic aldimines. Compounds **50** could be easily transformed successively into β-amino esters **51**, and the corresponding β-lactams **52** with high optical purity (Scheme 17) [89]. The absolute configurations of compounds **52** were obtained by the comparison of the signs of specific rotation of **52** with R = Ph(CH_2_)_2_, with that of known (*R*)-4-(2-phenylethyl)azetidin-2-one. A 6/4-fused bicyclic transition state model was proposed to rationalize the stereochemical outcome, in which the sodium metal is chelated by the oxygen and the nitrogen atoms of sulfinyl imine (Scheme 17), occurring the nucleophilic attack to the *Si* face of the imines with *R_S_* configuration.

**Scheme 18:**
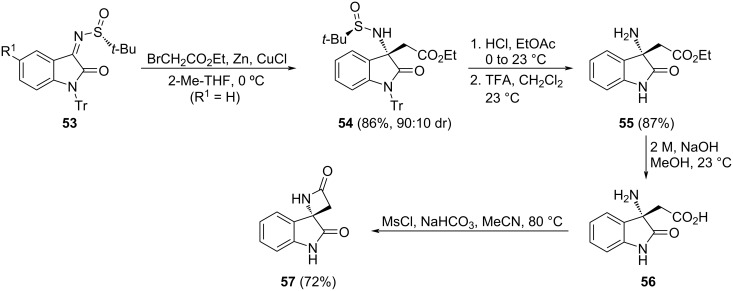
Synthesis of spiro-β-lactam **57** from chiral (*R*_S_)-*N*-*tert*-butanesulfinyl isatin ketimine **53** and ethyl bromoacetate.

Su and Xu reported the stereoselective synthesis of spiro β-lactam **57** from chiral (*R*_S_)-*N*-*tert*-butanesulfinyl isatin ketimine **53** (R^1^ = H), with a bulky trityl protecting group bonded to the nitrogen indolic atom (Tr = triphenylmethyl), and ethyl bromoacetate. The Zn/Cu-mediated Reformatsky-type reaction furnished enantiomerically pure compound **54** after column chromatographic purification. Selective desulfinylation of **54** was carried out by using 1.0 M HCl in EtOAc, and further removal of the Tr group by the employment of TFA in dichloromethane afforded the 2-oxoindolinyl amino ester derivative **55**, the key intermediate for the synthesis of (–)-AG-041R, a gastrin/cholecyctokinin-B receptor antagonist. In addition, amino ester **55** was transformed into the fully unprotected amino acid **56** under basic conditions in MeOH, and after that, further treatment with of MsCl and NaHCO_3_ in MeCN at 80 °C led to spiro-β-lactam derivative **57** in 72% combined yield (Scheme 18) [88].

**Scheme 19:**
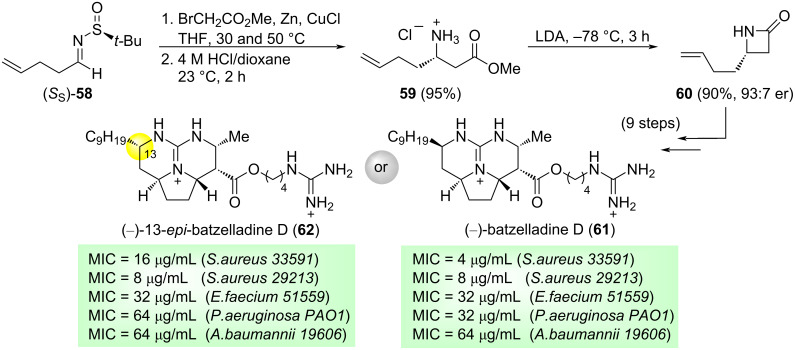
Synthesis of β-lactam **60**, a precursor of (−)-batzelladine D (**61**) and (−)-13-*epi*-batzelladine D (**62**) from chiral (*S_S_*)-*N*-*tert*-butanesulfinyl imine **58**, and antimicrobial evaluation of MCI values for these compounds.

In 2020, Pierce and co-workers developed a method for the synthesis of guanidinium alkaloid batzelladine D in enantiomeric and racemic form, along with a series of stereochemical analogues. The batzelladines are a family of polycyclic guanidinium alkaloids that were isolated in the mid-1990s from the Caribbean sponge *bataella* sp. From a biological point of view, the batzelladines have received attention due to their reported activity as inhibitors of HIV gp120-human CD4 binding. Chiral *N*-*tert*-butanesulfinyl aldimine (*S*_S_)-**58** was used as a precursor in the synthesis of (–)-batzelladine D **61** and (–)-13-*epi*-batzelladine D **62**. The reaction of (*S*_S_)-**58** with methyl bromoacetate in the presence on Zn and CuCl in THF, left, after removal of the sulfinyl group under acidic conditions, to β-amino ester ammonium chloride **59** in high yield. This compound was transformed into β-lactam **60** in 90% yield by treatment with LDA in THF at −78 °C [90]. Compound **60** was converted after 9 steps in target batzelladines D **61** and **62** (Scheme 19). The authors explored also the antimicrobial activity of these compounds against a series of pathogens with starting promising results.

### Asymmetric synthesis of pyrrolidines

The pyrrolidine ring is more represented within natural products than the 3- and 4-membered nitrogen-containing heterocycles. This molecular array is also found in drugs and other biologically active molecules. For this reason, there are numerous examples of synthetic methodologies for these compounds in the literature. In most cases, the pyrrolidine ring is formed from an amine with a hydrocarbon chain that also carries a functional group at the appropriate distance that allows the cyclization process to take place. In the case of substituted pyrrolidines, the stereoselective synthesis is especially interesting, highlighting the 1,3-dipolar cycloaddition reactions with azomethine ylides as an example of transformation that take place with great stereocontrol, and allow the synthesis of polyfunctionalized pyrrolidines in a single reaction step [91–92]. On the other hand, natural amino acids proline and hydroxyproline are functionalized pyrrolidines that have found great application in organic synthesis as chiral organocatalysts in stereoselective processes [93–94].

#### Cyclizations involving a position in the starting chiral imine

**Scheme 20:**
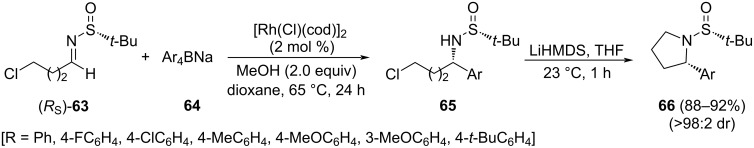
Rhodium-catalyzed asymmetric synthesis of 3-substituted pyrrolidines **66** from chiral imine (*R*_S_)-**63** and sodium tetraarylborates **64**.

Arylation of chiral sulfinyl imines with sodium tetraarylboronates **64** was found to proceed with high diastereoselectivity under rhodium catalysis. Reddy and co-workers applied this methodology to the synthesis of 2-substituted pyrrolidines **66** [95]. The arylation of chlorinated imine (*R*_S_)-**63** was performed with 2 mol % of an air-stable rhodium catalyst in dioxane, in the presence of 2 equivalents of MeOH, at 65 °C, leading to compounds **65** with high diastereomeric ratio. Crude amides **65** were converted into the corresponding pyrrolidines **66** in high yield by stirring at room temperature for 1 h in presence of 2.0 equivalents of LiHMDS (Scheme 20). It is important to highlight that cyclization occurred without epimerization with such as strong base.

**Scheme 21:**
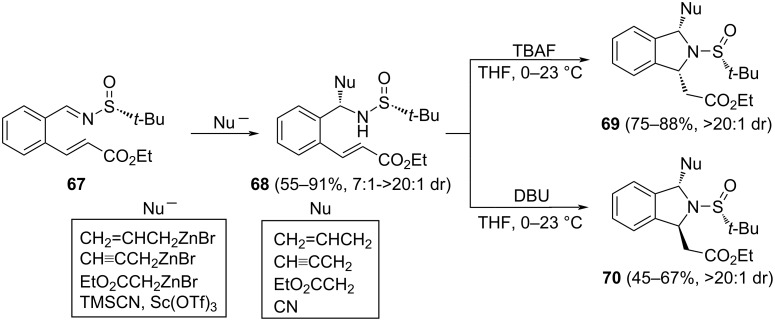
Asymmetric synthesis of 1,3-disubstituted isoindolines **69** and **70** from chiral imine **67**.

Isoindolines with substituents at 1 and 3 positions were synthesized from an aromatic *N*-*tert*-butanesulfinyl imine **67**, bearing a Michael acceptor in the *ortho*-position. Fustero, Barrio and co-workers found that combining an asymmetric nucleophilic addition to the chiral imine, with an intramolecular conjugate aza-Michael reaction, the expected 1,3-disubstituted isoindolines were produced with high diastereoselectivity [96]. Importantly, depending on the base involved in the intramolecular aza-Michael reaction, it was possible to reach either *cis*- or *trans*-isoindolines, **69** and **70**, respectively, from the same precursor **68**. The authors proposed that the thermodynamically more stable *cis*-isomer **69** is formed when TBAF was used. Meanwhile, working under kinetic conditions (DBU as base), *trans*-isomer **70** was obtained (Scheme 21).

**Scheme 22:**
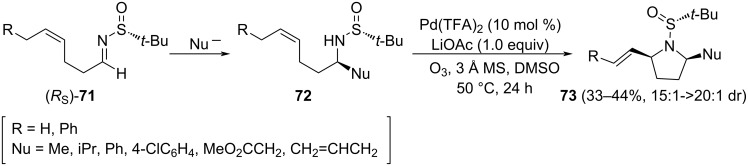
Asymmetric synthesis of *cis*-2,5-disubstituted pyrrolidines **73** from chiral imine (*R*_S_)-**71**.

The group of Stahl provided an elegant approach to the synthesis of 2,5-disubstituted pyrrolidines **73**, from alkenyl sulfinamides **72** [97]. These substrates were prepared from chiral *N*-*tert*-butanesulfinyl imine (*R*_S_)-**71**. Nucleophilic additions to this imine took place with high diastereoselectivity to the *Si* face of the iminic carbon. After that, the combination of Pd(TFA)_2_, lithium acetate and DMSO as solvent led to optimal results in the oxidative cyclization process to produce pyrrolidines **73** as a single diastereoisomer, with relative *cis*-configuration (Scheme 22). This has been the first reported nucleophilic attack of the sulfinyl group to a π-allylpalladium intermediate.

**Scheme 23:**
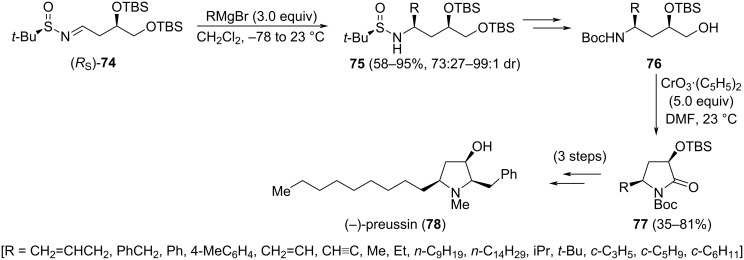
Asymmetric synthesis of 3-hydroxy-5-substituted pyrrolidin-2-ones **77** from chiral imine (R_S_)-**74**.

The asymmetric synthesis of 3-hydroxy-5-substituted pyrrolidin-2-ones **77**, with relative *cis*-configuration, was reported by the group of Huang and Wei [98]. A diastereoselective addition of Grignard reagents to chiral aldimine (*R*_S_)-**74**, and an intramolecular oxidative cyclization of aminoalcohols derivatives **76**, are key steps of this approach. Both diastereoisomers of aldimines **74** (*R*_S_ and *S*_S_) were prepared from ᴅ-malic acid and the corresponding enantiomer of *tert*-butanesulfinamide. Importantly, the choice of the solvent was crucial for obtaining high diastereoselectivities in the Grignard addition step, in which dichloromethane was performing better than THF. On the other hand, diastereoselectivities for addition products **75** were higher working with (*R*_S_)-**74** than its (*S*_S_) diastereoisomer, indicating a mismatch between the chiral auxiliary and the stereocenter in this substrate. Concerning the oxidative cyclization reaction, pyridinium dichromate (PDC) provided low yields of expected lactam **77**. Many oxidants were checked for this transformation to take place, and the Sarett reagent [CrO_3_·(C_5_H_5_)_2_] in DMF was the best to produce lactams **77**. The synthetic interest of these functionalized lactams was demonstrated in the synthesis of alkaloid (−)-preussin (**78**) from the appropriate precursor **77** in three steps (Scheme 23).

**Scheme 24:**
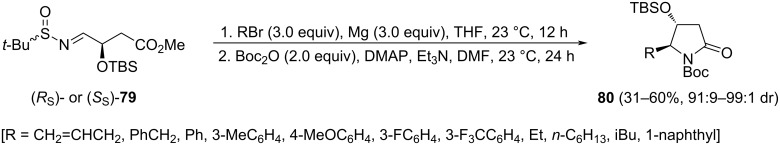
Asymmetric synthesis of 4-hydroxy-5-substituted pyrrolidin-2-ones **80** from chiral imines **79**.

Similar strategies were employed to obtain similar heterocycles [99]. Other regio- and stereoisomeric 2-pyrrolidones **80** were also prepared by a stereoselective tandem Barbier process of **79** with alkyl and aryl bromide [100]. The process was performed in this case under Barbier reaction conditions. Namely, the formation of the nucleophile (organomagnesium reagent) is carried out in the presence of the electrophile (chiral imine **79**). Surprisingly, both diastereomeric aldimines **79** (*R*_S_ and *S*_S_) gave similar results concerning the stereochemical outcome, suggesting that the chiral sulfonamide moiety was not involved in the stereocontrol during this tandem Barbier addition process. After addition of the organomagnesium reagent to the imine **79**, cyclization involving the magnesium amide and the ester occurred without the need of an extra cyclization step to give, after *N*-Boc protection, 4-hydroxy-5-substituted pyrrolidin-2-ones **90**, with relative *trans*-configuration (Scheme 24).

#### Cyclizations involving a position in the attacking nucleophile

**Scheme 25:**
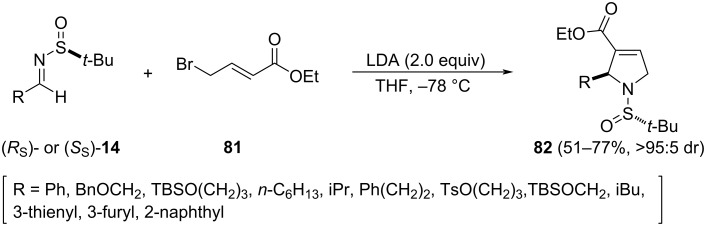
Asymmetric synthesis of 3-pyrrolines **82** from chiral imines **14** and ethyl 4-bromocrotonate (**81**).

The reaction of ethyl 4-bromocrotonate (**81**) with LDA at −78 °C and subsequent addition of chiral imines **14** afforded 3-pyrrolines **82** with high diastereoselectivity. Chogii and Njardarson proposed that after deprotonation of **81**, the resulting dienolate reacted at α-position with the chiral imine **14**. The addition was highly diastereoselective, being the configuration of the newly created stereogenic center dependent on the configuration of the sulfur atom of the starting imine **14**. After nucleophilic addition, and subsequent elimination, 3-pyrrolines **82** were formed as single diastereomers [101]. The whole process could be considered a [3 + 2] annulation, and aziridines were not observed as competing reaction products (see above Scheme 11). In addition, hindered imines, ethers, sulfonates, heteroaryl substituents, and conjugated imines were all well tolerated (Scheme 25).

**Scheme 26:**
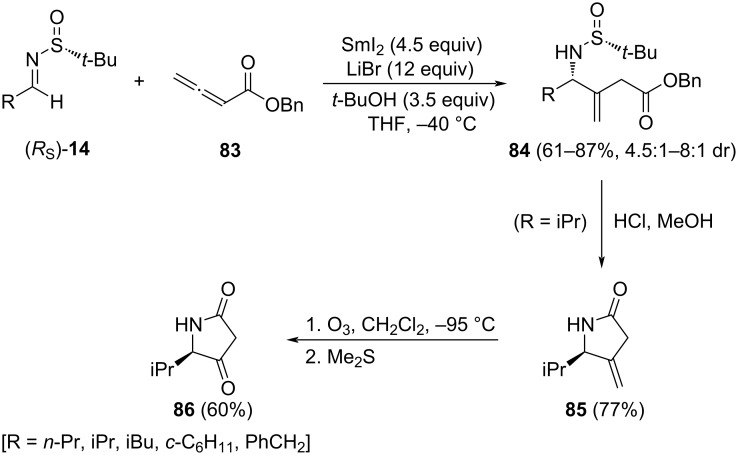
Asymmetric synthesis of γ-amino esters **84**, and tetramic acid derivative **86** from chiral imines (*R*_S_)-**14** and allenoate **83**.

A SmI_2_-mediated coupling of allenoate **83** with chiral (*R*_S_)-**14** provided γ-amino ester derivatives **84** in good yields and moderate diastereomeric ratios. Huang and Py found that the better yields and diastereoselectivities were found working in THF as solvent in the presence of *t*-BuOH and LiBr as additives [102]. The isopropyl-substituted derivative **84** was easily converted into the corresponding methylene lactam **85**, upon removal of the sulfinyl unit under acidic conditions. Finally, ozonolysis of **85** yielded tetramic acid **86** in 60% yield (Scheme 26). The configuration of the newly generated chiral center in compounds **84** was assigned from the sign of the optical rotation of enantioenriched tetramic acid **86**, which was previously characterized. Based on this, it can be stated that addition of the allenoate **83** takes place mainly to the *Re* face of imines with (*R*_S_)-configuration.

**Scheme 27:**
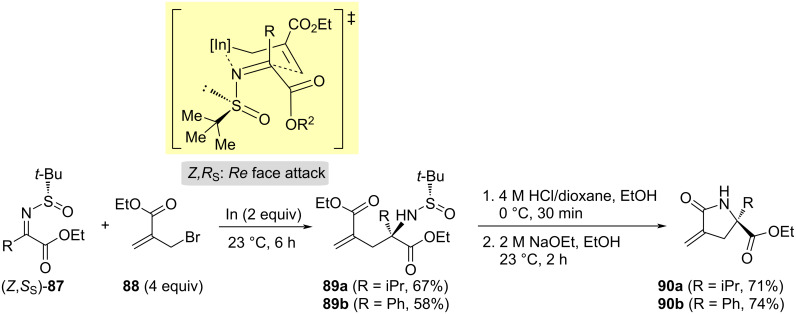
Asymmetric synthesis of α-methylene-γ-butyrolactams **90** from chiral imines (*Z*,*S*_S_)-**87** and ethyl 2-bromomethylacrylate **88**.

Excellent diastereoselectivities were also achieved in the indium-mediated allylation of chiral *tert*-butanesulfinyl glyoxylate imine derivatives **87** with ethyl 2-bromomethylacrylate (**88**). Working at room temperature without any additional solvent provided the highest yields in these coupling reactions, amino diesters **89** being isolated as single diastereoisomers (Scheme 27). Removal of the sulfinyl group under acidic conditions, and further treatment of the resulting ammonium salts with sodium ethoxide, yielded α-methylene-γ-butyrolactams **90**, in a one-pot, two-step process [103]. A six-membered chair-like transition state model with the indium coordinated to the nitrogen atom of the imine, and the sulfinyl and R groups located at axial positions, in a kind of s-*cis* conformation, was proposed to rationalize the stereochemical outcome. By considering this working model, the nucleophilic attack took place to the *Re* face of imines with (*Z,S*_S_)-configuration (Scheme 27).

**Scheme 28:**
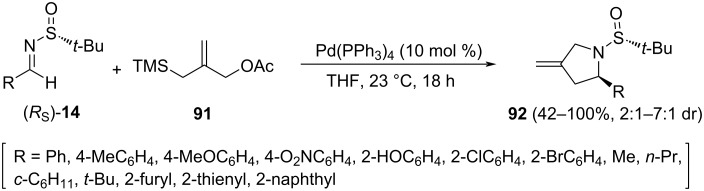
Asymmetric synthesis of methylenepyrrolidines **92** from chiral imines (*R*_S_)-**14** and 2-(trimethysilylmethyl)allyl acetate **91**.

The cycloaddition of chiral sulfinyl imines (*R*_S_)-**14** with 2-(trimethysilylmethyl)allyl acetate (**91**) could also be promoted by Pd(0) to give methylenepyrrolidines **92**. The group of Stockman demonstrated that Pd(PPh_3_)_4_ was the best source of Pd(0) and that the reaction worked well in different solvents, with dry THF giving the best diastereoselectivities and good yields at room temperature [104]. The configuration of the major diastereoisomer was assigned by X-ray crystallographic analysis. From this, authors rationalized the stereochemical outcome of the cyclization considering that the stereoinduction is derived from the dipole–dipole repulsion of the sulfinyl imine, which places the *tert*-butyl group on the *Si* face, and thus the cycloaddition occurs from the less sterically hindered *Re* face. The cyclization process worked also in *tert*-butanesulfinyl ketimines, but yields and diastereoselectivities were significantly lower (Scheme 28).

**Scheme 29:**
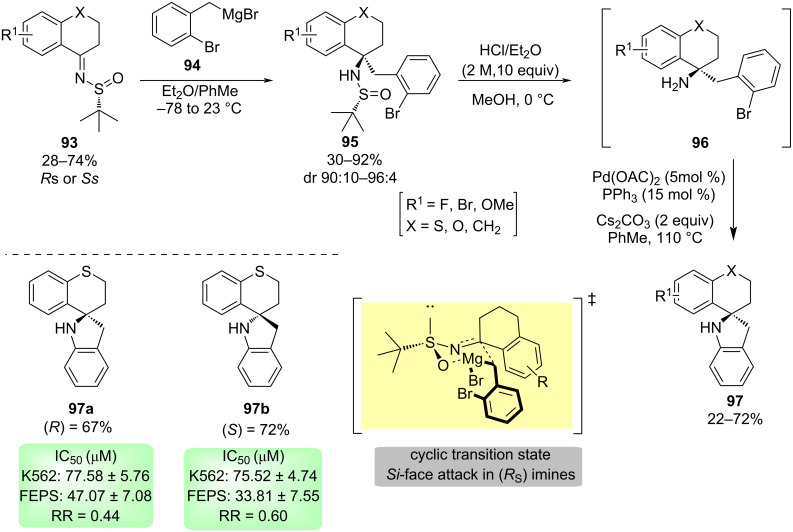
Synthesis of dibenzoazaspirodecanes from cyclic *N*-*tert*-butanesulfinyl imines.

Recently, a series of alkaloids like dibenzoazaspirodecanes **97** have been synthesized by addition of 2-bromobenzylmagnesium bromide (**94**) to chiral *N-tert*-butanesulﬁnyl imines **93**. These reactions proceeded with high levels of diastereocontrol. The resulting sulfonamide derivatives **95** were transformed into the target spiro compound **97** by performing successive desulfinylation and intramolecular palladium-catalyzed *N*-arylation. To rationalize the stereochemical course of the addition, DFT calculations were performed and they predicted correctly the observed experimental results considering a six-membered ring cyclic transition state. The addition took place to the *Si*-face of the imines with (*R*_S_)-configuration. Compounds **97** were also evaluated in a preliminary study in leukemia strains (Scheme 29) [105].

**Scheme 30:**
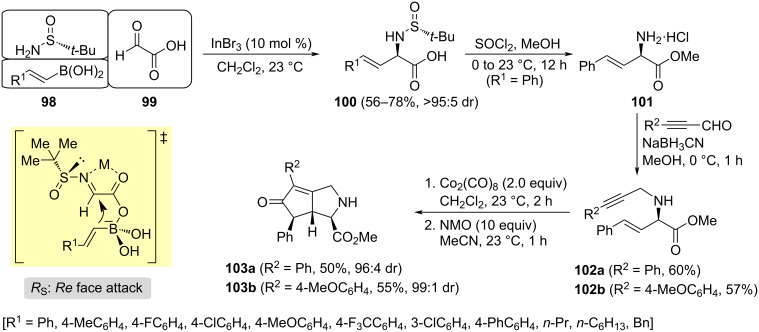
Stereoselective synthesis of cyclopenta[*c*]proline derivatives **103** from β,γ-unsaturated α-amino acids **100**.

Li and Xu reported a method for the enantioselective synthesis of β,γ-unsaturated α-amino acids **100**, by a Lewis acid-promoted diastereoselective Petasis reaction of vinylboronic acids **98**, (*R*)-*N-tert*-butanesulfinamide and glyoxylic acid (**99**). They found that the best results were obtained working with InBr_3_ as Lewis acid, in dichloromethane at room temperature [106]. Under these reaction conditions, sulfinyl imine is formed first along with the boronate by interaction of the corresponding vinylboronic acid with the carboxylic group of the imino acid intermediate. The transfer of the vinyl unit to the electrophilic iminic carbon took place in a quite rigid system, with chelation of the Lewis acid with the nitrogen of the imine and carboxylate oxygen, forming a five-membered ring. The migration of the vinyl group occurred to the *Re* face of the imine, which is less shielded than the *Si* face, because of the influence of the bulky *tert*-butyl in the most stable conformation of the sulfinyl imine (Scheme 30). The authors also demonstrated the synthetic utility of compounds **100**. Their reaction with thionyl chloride in methanol produced removal of the sulfinyl group and formation of the corresponding methyl ester, to give compounds **101**. Subsequent, reductive ammination with 3-phenyl-2-propynal led to reaction intermediates **102**, which under typical Pauson–Khand reaction conditions gave cyclopenta[*c*]proline derivatives **103** in moderate yields, with high diastereoselectivities (Scheme 30).

### Asymmetric synthesis of piperidines

The six-membered nitrogen-containing rings are the most common heterocycles among natural products and also synthetic pharmaceutical drugs [107–108]. For this reason, the piperidine unit has attracted great attention among organic chemists [109]. Due to that, a large number of classical methodologies have been used for their synthesis, in which the key step is the generation of the six-membered ring, including the aldol reaction, the reductive amination, Mannich reaction, ring closing metathesis, Diels–Alder reaction with imines as dienophiles, aza-Prins cyclization, and intramolecular Michael reaction, among others [110–115]. Despite the considerable effort made to date in this field, the development of new methodologies that allow accessing these heterocycles in a stereoselective way, and taking into account environmental considerations as one of the most important points, continue to be of great interest [109,116–118].

#### Cyclizations involving a position in the starting chiral imine

**Scheme 31:**
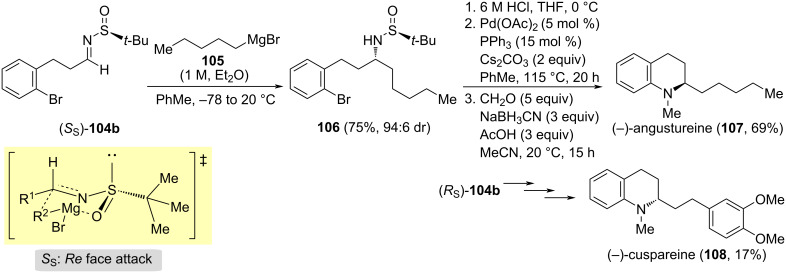
Stereoselective synthesis of alkaloids (−)-angustureine (**107**) and (−)-cuspareine (**108**).

Enantiomeric *N*-*tert*-butanesulfinyl imines **104b** derived from 3-(2-bromophenyl)propanal have been used as reaction intermediates in the synthesis of tetrahydroquinoline alkaloids (−)-angustureine (**107**) and (−)-cuspareine (**108**) reported by Sirvent et al. [119]. The diastereoselective addition of a Grignard reagent was a key step in this methodology. The addition proceeded with high diastereoselectivity in toluene, and the attack of the Grignard reagent occurred on the *Re* face of the imine with *S* configuration at the sulfur atom, through a chelated transition state. The reaction of chiral aldimine (*S*_S_)-**104b** with pentylmagnesium bromide gave compound **106** in 75% yield. Further successive *N*-desulfinylation, intramolecular palladium-catalyzed *N*-arylation, and final *N*-methylation led to (−)-angustureine (**107**) in high overall yield (Scheme 31). The same methodology was applied to the synthesis of (−)-cuspareine (**108**), starting in this case from enantiomeric imine (*R*_S_)-**104b**, and using 2-(3,4-dimethoxyphenyl)ethylmagnesium bromide as Grignard reagent.

**Scheme 32:**
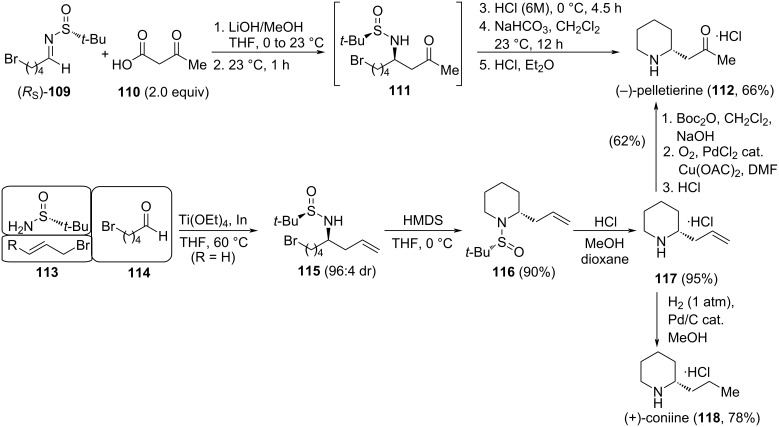
Stereoselective synthesis of alkaloids (−)-pelletierine (**112**) and (+)-coniine (**117**).

A straightforward synthesis of the alkaloid (−)-pelletierine (**112**) was accomplished by the diastereoselectivity coupling of 3-oxobutanoic acid (**110**) and the *N*-*tert*-butanesulfinyl imine (*R_S_*)-**109** derived from 5-bromopentanal (**114**). The base-promoted decarboxylative-Mannich coupling of these reagents led to β-amino ketone derivative **111**, which was not isolated. After removal of the sulfinyl group under acidic conditions, and intramolecular N-alkylation upon treatment with sodium bicarbonate, (−)-pelletierine (**112**) was formed, and easily isolated as its hydrochloride derivative (Scheme 32) [120]. Compound **112** is a key intermediate in the biomimetic synthesis of natural alkaloids. Interestingly, amino allylation of 5-bromopentanal (**114**) with (*R*)-*tert*-butanesulfinamide and allyl bromide (**113**, R = H) in the presence of indium metal gave homoallylamine derivative **115**. In this transformation, imine (*R_S_*)-**109** is a reaction intermediate that was not isolated. Treatment of **115** with potassium hexamethyldisilazide (KHMDS) led to the sulfinyl piperidine derivative **116**, and final deprotection under acidic conditions produced enantioenriched 2-allylpiperidine (**117**) as its hydrochloride (Scheme 32) [121]. Compound **117** has been also an advanced intermediate in the synthesis of alkaloids. For instance, hydrogenation of **117** yielded (+)-coniine (**118**), the major alkaloid extracted from poison hemlock and responsible for its toxicity, as its hydrochloride, and *N*-Boc protected derivative of **117** submitted to Wacker oxidation led to (−)-pelletierine (**112**) alkaloid.

**Scheme 33:**
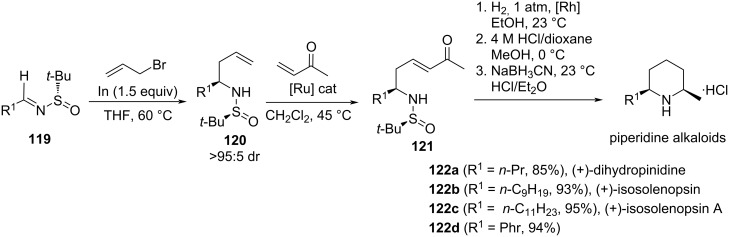
Synthesis of piperidine alkaloids (+)-dihydropinidine (**122a**), (+)-isosolenopsin (**122b**) and (+)-isosolenopsin A (**122c**) .

Yus and co-workers developed the synthesis of the piperidine alkaloids (+)-dihydropinidine (**122a**), (+)-isosolenopsin (**122b**), (+)-isosolenopsin A (**122c**) and (2*R*,6*R*)-6-methylpipecolic acid hydrochloride by oxidation of the aromatic ring of (2*R*,6*R*)-2-methyl-6-phenylpiperidine (**122d**). The diastereoselective allylation of (*S*_S_)-*N-tert*-butanesulfinyl imines **119** mediated by indium metal under Barbier's reaction connections (formation of the allylindium intermediate in the presence of the imine electrophile) is the key step in these syntheses (*Re*-face attack). The natural products were obtained after four additional steps: cross-metathesis of allylated compounds **120** with methyl vinyl ketone, reduction of conjugated C=C double bond, removal of the sulfinyl group under acidic conditions, and final stereoselective reduction of the imine formed by intramolecular cyclization (Scheme 33) [122].

**Scheme 34:**
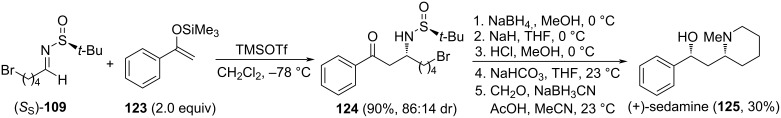
Stereoselective synthesis of the alkaloids(+)-sedamine (**125**) from chiral imine (*S*_S_)-**119**.

The group of Prasad reported the diastereoselective synthesis of β-amino ketone derivatives from *N*-*tert*-butanesulfinyl imines and silyl enol ethers of aryl methyl ketone [123]. The synthetic interest in β-amino ketones was exemplified in the synthesis of alkaloid (+)-sedamine (**125**), which has been shown to display memory-enhancing properties and was also effective for the treatment of cognitive disorders. The reaction of the *N*-*tert*-butanesulfinyl imine (*S*_S_)-**119** with trimethylsilyl enol ether derived from acetophenone **123** in the presence of TMSOTf at low temperature, produced β-amino ketone derivative **124** in high yield and diastereoselectivity (Scheme 34). A reduction of **124** gave a mixture of diastereomeric alcohols, and the one with (*R*)-configuration at the benzylic position was isolated in 54% yield. Further treatment of the alcohol with NaH furnished a cyclized product, which after desulfination and *N*-methylation led to expected (+)-sedamine (**125**) in 30% overall yield from ketone derivative **124**.

**Scheme 35:**
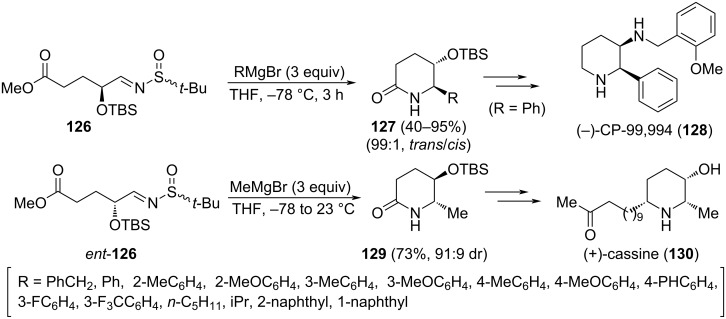
Stereoselective synthesis of *trans*-5-hydroxy-6-substituted-2-piperidinones **127** and **129** from chiral imines **126**.

The stereoselective synthesis of *trans*-5-hydroxy-6-substituted-2-piperidinones was also reported by the group of Wei, taking advantage of the addition of Grignard reagents to *N*-*tert*-butanesulfinyl α-alkoxy aldimines **126** [124]. In this one-pot approach, a successive nucleophilic addition–cyclization–desulfinylation took place, leading directly to piperidinones **127**. The reactions were performed in THF at −78 °C for 3 hours. Yields ranged from moderate to excellent with aliphatic and aromatic organomagnesium compounds. Based on X-ray crystallographic analyses, the relative configurations of the products **127** were unambiguously assigned as *trans*-form. The stereocontrol was governed by the stereogenic center bearing the OTBS group at α-position of the imine, showing no influence on it the configuration of the sulfur atom of the sulfinyl unit. This methodology was applied to the asymmetric synthesis of (−)-CP-99,994 (**128**), the enantiomer of a promising clinical agent which displays a variety of biological activities, including neurogenic inflammation, pain transmission, and regulation of the immune response. Starting from α-alkoxy aldimines *ent*-**126**, the utility of this methodology was also demonstrated in the synthesis of alkaloid (+)-cassine (**130**), isolated from the leaves and twigs of *Cassia excelsa*, displaying antimicrobial activity. Methylmagnesium bromide was the Grignard reagent in this synthesis, with a 18% overall yield after seven steps from aldimine *ent*-**126** (Scheme 35) [125].

**Scheme 36:**
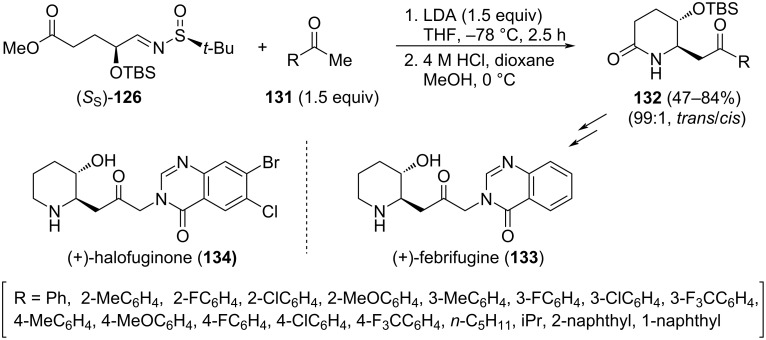
Stereoselective synthesis of *trans*-5-hydroxy-6-substituted ethanone-2-piperidinones **132** from chiral imine (*S*_S_)-**126**.

The reaction of chiral α-siloxyl imine (*S*_S_)-**126** with enolates derived from methyl ketones **131** was also investigated. The enolate was formed with LDA at −78 °C and reacted at the same temperature with imine (*S*_S_)-**126** for 2.5 hours. The addition proceeded with high diastereoselectivity, followed by cyclization. Final acid treatment produced the removal of the sulfinyl group leading to *trans*-5-hydroxy-6-substituted ethanone-2-piperidinones **132** in moderate to high yields, as a single diastereoisomer [126]. The diastereoselectivity of the addition was controlled in this case by both the α-siloxyl group and the chiral sulfinamide moiety. Interestingly, the utility of this approach was also demonstrated by the synthesis of (+)-febrifugine (**133**), a natural product isolated from Chinese medicinal plants *Dichroa febrifuga* Lour., and (+)-halofuginone (**134**), which is a pharmaceutical candidate developed from febrifugine for the treatment of human scleroderma (Scheme 36).

**Scheme 37:**
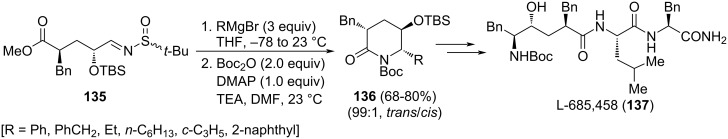
Stereoselective synthesis of *trans*-3-benzyl-5-hydroxy-6-substituted-2-piperidinones **136** from chiral imines **137**.

The reaction of chiral imine **135** with Grignard reagents in THF took also place with high diastereoselectivity. Starting imine **135** with two well-defined stereogenic centers at the hydrocarbon backbone were prepared as a mixture of (*R*_S_)- and (*S*_S_)-diastereoisomers from ᴅ-glutamic acid. After nucleophilic addition to the imine, a successive cyclization–desulfinylation occurred to give the corresponding piperidinone. Final reaction with di-*tert*-butyl dicarbonate led to functionalized 2-piperidinones **136** [127]. These compounds are interesting reaction intermediates because they can be transformed by conventional reactions into, for instance, compound L-685,458 (**137**), an inhibitor of γ-secretase, with potential interest for the treatment of Alzheimer’s disease and other neurological disorders (Scheme 37).

**Scheme 38:**
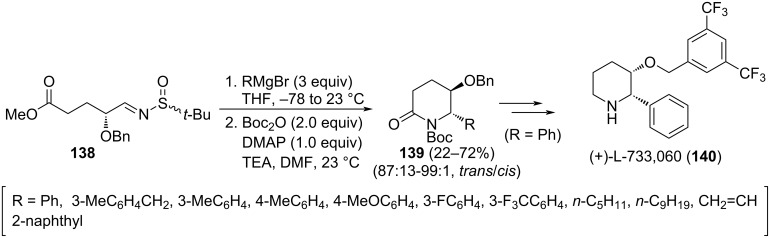
Stereoselective synthesis of *trans*-5-hydroxy-6-substituted 2-piperidinones **139** from chiral imine **138**.

The diastereoselective synthesis of *trans*-5-hydroxy-6-substituted 2-piperidinones **139** was also achieved from *O*-benzyl protected aldimine **138** following the previously commented tandem Grignard reagent addition, subsequent cyclization-desulfinylation, and final *N*-Boc protection. The stereochemical pathway is controlled exclusively again by the configuration of the stereogenic center bearing the benzyloxy group [128]. Interestingly, chiral δ-lactams **139** are synthetic intermediates that can be transformed into compound (+)-L-733,060 (**140**), a potent neurokinin substance P receptor antagonist. This compound displays a wide variety of biological activities, including inhibition of neurogenic inflammation, blocking of pain transmission and regulation of immune response (Scheme 38).

**Scheme 39:**
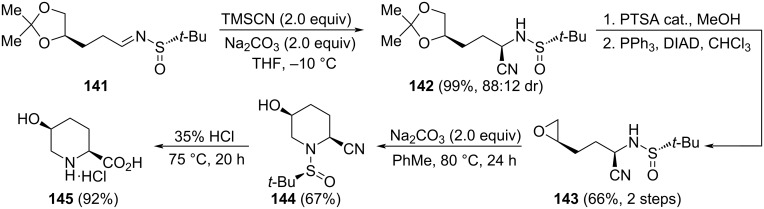
Stereoselective synthesis of ʟ-hydroxypipecolic acid **145** from chiral imine **144**.

A stereoselective synthesis of ʟ-hydroxypipecolic acid **145** was reported recently by Zhang and Sun. Compound **145** is an intermediate for the synthesis of β-lactamase inhibitors. A key step in this synthesis was the hydrocyanation of chiral sulfinyl imine **141**, prepared from commercially available and inexpensive ʟ-glyceraldehyde acetal, with trimethylsilyl cyanide (TMSCN) in THF at −10 °C. The reaction product **142** was obtained in quantitative yield and good diastereomeric ratio. Further hydrolysis of the cyclic acetal, and subsequent epoxidation of the resulting diol under typical Mitsunobu conditions led to epoxide derivative **143**. The piperidine ring was formed through a 6-*endo*-*tet* cyclization by treatment of the epoxide **143** with sodium carbonate in toluene at 80 °C. Hydrolysis of the cyano group under acidic conditions of compound **144** led to expected ʟ-hydroxypipecolic acid hydrochloride **145** in high yield (Scheme 39) [129].

**Scheme 40:**
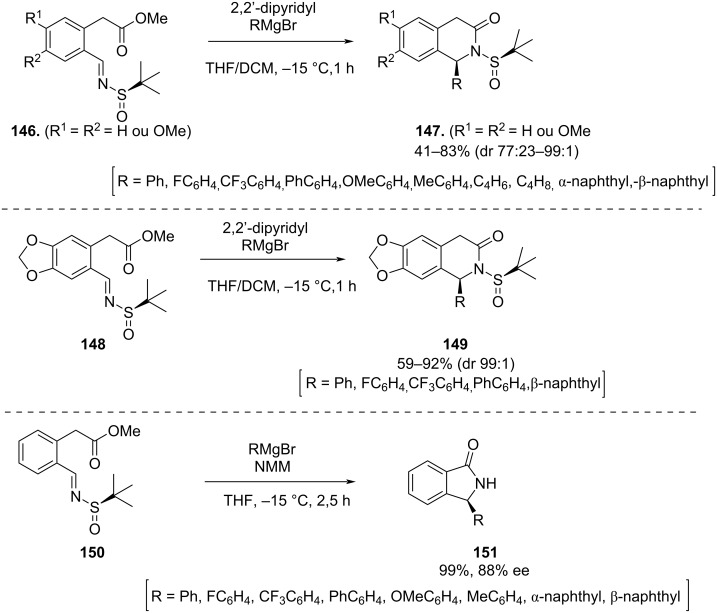
Synthesis of 1-substituted isoquinolones **147**, **149** and **151**.

In 2018, Wei and co-workers described the diastereoselective synthesis of 1-substituted isoquinolones using one-pot addition–cyclization–deprotection of the imine with Grignard reagents [130]. In this work, the addition to chiral imines **146**, **148** and **150** was performed using 2,2’-dipyridyl- or 4-methylmorpholine (NMM) to promote the complexation with the Grignard reagent. Products **147** and **149** were obtained in excellent yields and high diastereoselectivity and when 4-methylmorpholine (NMM) was used as additive, the heterocycle **151** was obtained in one pot addition–cyclization–deprotection of imine **150** (Scheme 40).

**Scheme 41:**
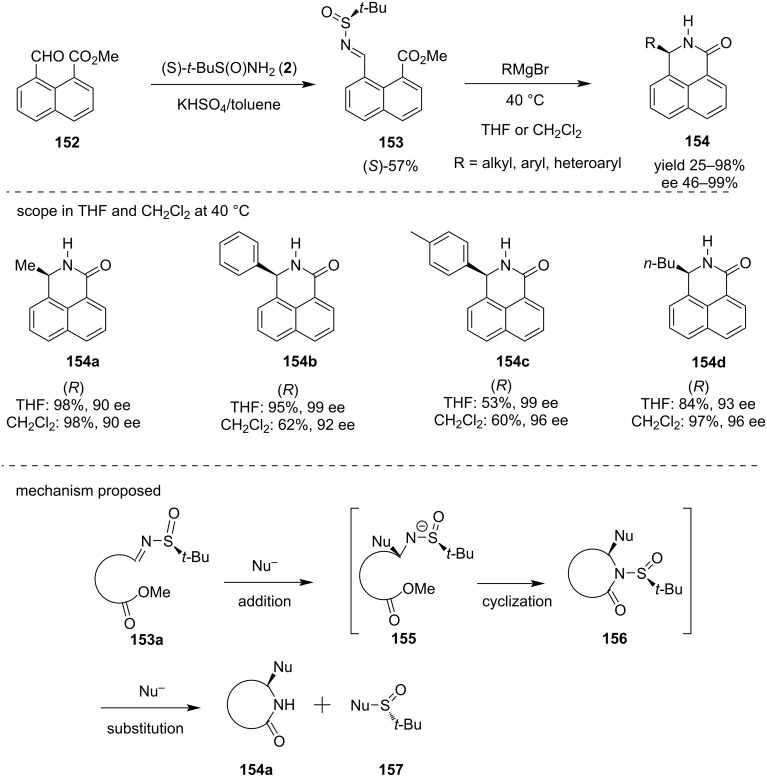
Stereoselective synthesis of 3-substituted dihydrobenzo*[de]*isoquinolinones **154**.

In 2020, Kaczorek and Kawęcki described the stereoselective synthesis of 3-substituted dihydrobenzo[*de*]isoquinolones **154** in both enantiomeric forms in one step. In this study, they reported an addition–cyclization–substitution reaction employing (*Rs*) and (*Ss*) *N*-*tert*-butylsulfinyl imine **14** and Grignard reagents using THF or CH_2_Cl_2_ as solvent at 40 °C. The 3-substituted dihydrobenzo[*de*]isoquinolinones **154** were obtained with good yield and with enantiomeric excess of 46–99%. The mechanism was explained by stereoselective addition of the Grignard reagent to the *N*-sulfinyl imine **153a** derived from **152**, in a subsequent cyclization to obtain the intermediate **156** then, a substitution at the sulfur atom occurred to form **154a** and **157** [131] (Scheme 41).

**Scheme 42:**
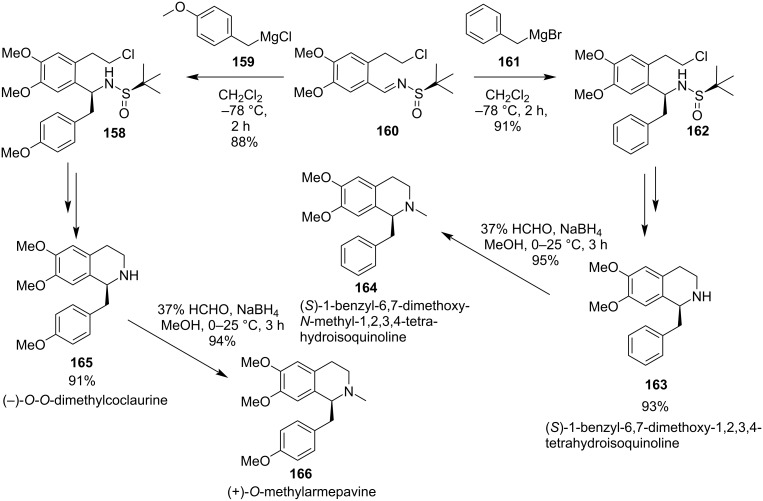
Enantioselective synthesis of alkaloids (S)-1-benzyl-6,7-dimethoxy-1,2,3,4-tetrahydroisoquinoline (**163**), (S)-1-benzyl-6,7-dimethoxy-*N*-methyl-1,2,3,4-tetrahydroisoquinoline (**164**), (−)-*O*,*O*-dimethylcoclaurine (**165**) and (+)-*O*-methylarmapavine (**166**).

In 2017, Reddy and co-workers described the stereoselective synthesis of (*S*)-1-benzyl-6,7-dimethoxy-1,2,3,4-tetrahydroisoquinoline (**163**), (*S*)-1-benzyl-6,7-dimethoxy-*N*-methyl-1,2,3,4-tetrahydroisoquinoline (**164**), (−)-*O,O*-dimethylcoclaurine (**165**) and (+)-*O*-methylarmapavine (**166**) alkaloids via chiral *tert*-butylsulfinamide through a haloamide cyclization. The strategy was based on the addition of organomagnesium bromide or chloride to chiral *N*-sulfinyl imine **160**. A subsequent base promoted cyclization of chloroamides (**158** and **162**) and the products **165** and **163** were obtained in 91% and 93% yields respectively. The *N*-methylation of alkaloids **163** and **165** using 37% formaldehyde and sodium borohydride formed the tetrahydroisoquinoline **164** and **166** in high yields of 95% and 94%, respectively [132] (Scheme 42).

**Scheme 43:**
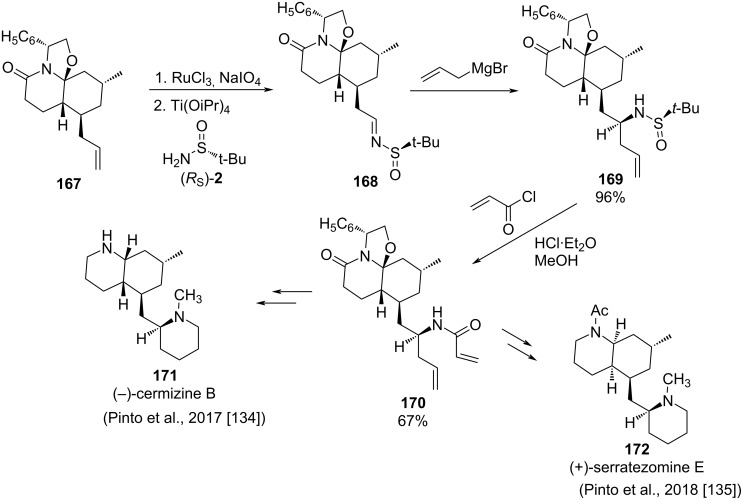
Enantioselective synthesis of alkaloids (−)-cermizine B (**171**) and (+)-serratezomine E (**172**) developed by Pinto and co-workers.

Pinto and co-workers reported recently the enantioselective synthesis of natural alkaloids (−)-cermizine B **171** and (+)-serratezomine E **172**. A key step of the synthetic strategy is the allylation with allylmagnesium bromide of *N*-*tert*-butanesulfinyl imine **168**. At the allylation step, a new chiral center with *S* configuration in compound **169** is formed in 96% yield. After removal the chiral sulfinyl group under acid conditions, treatment with acryloyl chloride produced acrylamide derivative **170**. From this common intermediate, and after several subsequent steps, including ring-closing metathesis [133], (−)-cermizine B (**171**) and (+)-serratezomine E (**172**) were obtained 57% and 72% yield, respectively (Scheme 43) [134–135].

#### Cyclizations involving a position in the attacking nucleophile

**Scheme 44:**
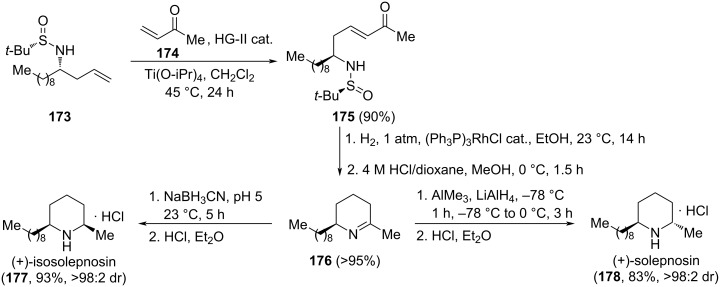
Stereoselective synthesis of (+)-isosolepnosin (**177**) and (+)-solepnosin (**178**) from homoallylamine derivative **173**.

Isosolenopsin (**177**) and solenopsin (**178**) are two isomeric piperidine alkaloids isolated from fire ants (*Solenopsis*) and display hemolytic, insecticide and antibiotic properties. A straightforward synthesis of these natural products from a common imine intermediate was reported by Medjahdi et al. comprising as key steps the indium–titanium-mediated aminoallylation of nonanal with (*R*)-*tert*-butanesulfinamide and allyl bromide, giving rise homoallylamine derivative **173**, a subsequent cross metathesis with methyl vinyl ketone (**174**) catalyzed by Hoveyda–Grubbs second generation catalyst to produce compound **175**, followed by hydrogenation–desulfynation, and final stereoselective reduction of the resulting cyclic imine intermediate **176**. In this diastereodivergent approach, reduction of this imine with sodium borohydride in a citrate-phosphate buffer medium (pH 5) led to (+)-isosolenopsin (**177**) in 93% yield and >98:2 *cis*/*trans* selectivity. On the other hand, when the reduction of imine **177** was carried out applying H. Yamamoto’s protocol (AlMe_3_/LiAlH_4_), (+)-solenopsin (**178**) was isolated in 83% yield and with excellent diastereoselectivity (>98:2 *trans*/*cis* selectivity, Scheme 44) [136].

**Scheme 45:**
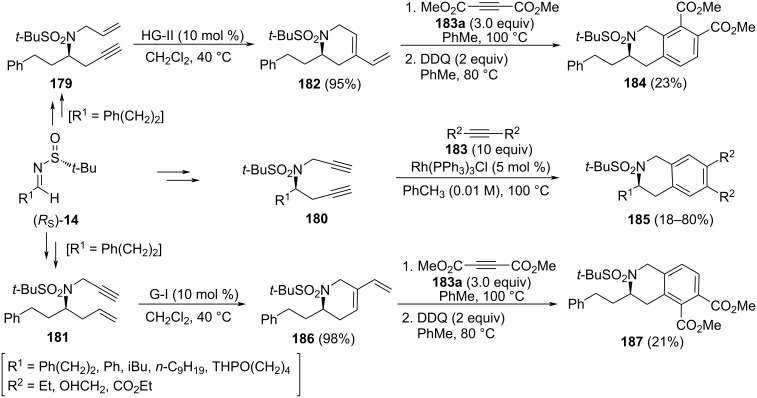
Stereoselective synthesis of tetrahydroquinoline derivatives **184**, **185** and **187** from chiral imines (*R*_S_)-**14**.

There are many compounds with a 1,2,3,4-tetrahydroisoquinoline structural motif bearing substituents at 1-position which display a wide range of biological activities. However, compounds bearing substituents at 3-position are less represented in nature and among pharmaceutical drugs. A multistep methodology to synthesize 1,2,3,4-tetrahydrosioquinolines **185** bearing substituents at 3-, 6- and 7-positions in a highly enantioselective fashion starting from chiral *N*-*tert*-butanesulfinyl imines (*R*_S_)-**14** was reported by Sirvent et al. [137]. The key step of this synthesis is a [2 + 2 + 2] cyclotrimerization by means of Wilkinson catalyst of azadiyne system **180**, which was accessible from imine **14** by consecutive diastereoselective indium-promoted propargylation, selective *N*-propargylation and final oxidation of the sulfinyl group. Sulfinyl imines (*R*_S_)-**14** could be also precursors of tetrahydroisoquinolines with substituents at different positions of the aromatic ring, by combining allylation and propargylation processes as the first steps of this new strategy. The resulting azaenynes **179** and **181** were efficiently transformed by a ruthenium-catalyzed ring-closing metathesis into cyclic 1,3-dienes **182** and **186**, respectively. The best results were obtained by performing the metathesis with Hoveyda–Grubbs second generation catalyst in the synthesis of cyclodiene **182**, and with Grubbs first generation catalyst for compound **186**. When these dienes reacted with dimethyl acetylenedicarboxylate in toluene at 100 ºC, followed by dehydrogenation of the resulting [4 + 2] adduct with DDQ, the expected 7,8- or 5,6-bis(methoxycarbonyl)substituted 1,2,3,4-tetrahydrosioquinolines **184** and **187**, were obtained, respectively (Scheme 45).

**Scheme 46:**
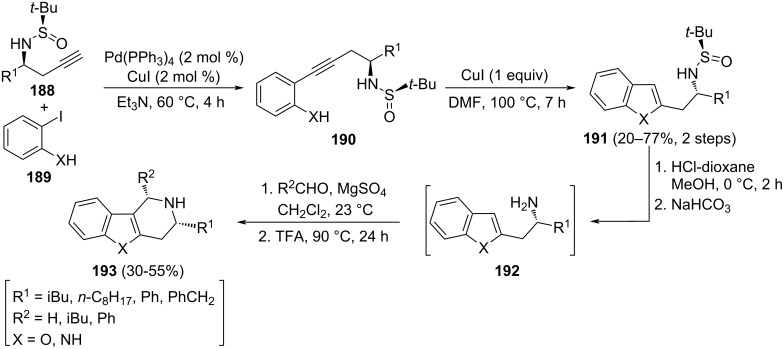
Stereoselective synthesis of pyridobenzofuran and pyridoindole derivatives **193** from homopropargylamine derivatives **192**.

Many indole alkaloids have been known for years and used in ancient cultures as psychotropic, stimulants and poisons. On the other hand, benzofurans and indoles, with a 2-aminoalkyl substituent at the 2-position, are not common compounds nor are they represented in nature. For that reason, synthetic methodologies to access these systems are of interest in order to explore their biological activity. Homopropargylamine derivatives **188** were obtained in a highly diastereoselective fashion (>95:5 dr) by nucleophilic addition of allenylindium intermediate to chiral *N*-*tert*-butanesulfinyl imines **14**. Subsequent Sonogashira coupling of compounds **188** with *o*-iodophenol (X = O) or *o*-iodoaniline (X = NH) **189**, led to 2-(2-aminoalkyl)benzofuran (X = O) and -indole (X = NH) derivatives **191**. Further removal of the sulfinyl unit under acidic conditions produced amine derivatives **192**, which were transformed into tetrahydropyrido-benzofuran (X = O) and indole (X = NH) derivatives **193** with relative *cis*-configuration, upon reaction with aldehydes. This Pictet–Spengler condensation was facilitated by the nucleophilic character of the 3-position of the benzofuran or indole moiety (Scheme 46) [138].

**Scheme 47:**
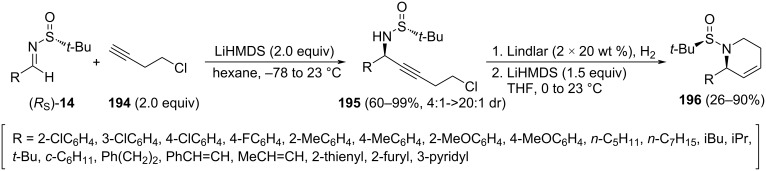
Stereoselective synthesis of 2-substituted 1,2,5,6-tetrahydropyridines **196** from chiral imines (*R*_S_)-**14**.

Turlington and co-workers reported a stereoselective synthesis in three steps of 2-substituted 1,2,5,6-tetrahydropyridines **196** starting from chiral *N*-*tert*-butanesulfinyl imines (*R*_S_)-**14** [139]. The synthesis commenced with addition of the organolithium compound resulting from the deprotonation of 4-chloro-1-butyne (**194**) to the imine. The propargylamine derivatives **195** were obtained in high yields and diastereoselectivities (>20:1 dr, in most cases). The lowest diastereoselectivity was found for pyridyl-substituted imine **14** (R = 3-pyridyl, 4:1 dr), due probably to competitive coordination of the lithium acetylide by the heteroatoms present in these imines. Reduction of the triple bond with Lindlar catalyst to provide olefin with *cis*-configuration, and cyclization using LiHMDS led to 1,2,5,6-tetrahydropyridines **196** (Scheme 47).

**Scheme 48:**
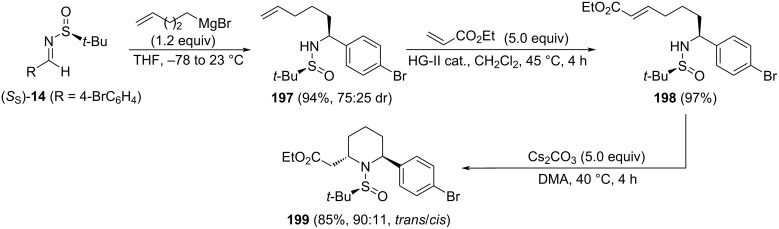
Stereoselective synthesis of 2-substituted trans-2,6-disubstituted piperidine **199** from chiral imines (S_S_)-**14** (R = 4-BrC_6_H_4_).

Many methods have been provided to generate thermodynamically stable *cis*-2,6-disubstituted piperidines, but the synthesis of *trans*-derivatives remains elusive. In this regard, Bhattacharjee and co-workers reported a highly efficient large-scale synthesis of 2,6-*trans*-piperidine derivative **199** from easily available starting material [140]. Key steps of the synthesis are the diastereoselective addition of 4-pentenylmagnesium bromide to chiral imine (*S*_S_)-**14** (R = 4-BrC_6_H_4_). Two diastereoisomers **197** were isolated in 94% yield and moderate diastereoselectivity. The major component of the mixture was transformed into compound **198** through a Hoveyda–Grubbs second generation cross metathesis with ethyl acrylate. Another key step was the intramolecular aza-Michael reaction promoted by cesium carbonate as base in dimethylacetamide (DMA), leading to 2,6-*trans*-piperidine derivative **199** in high yield (Scheme 48). This compound was an intermediate in the synthesis of a novel class of anti-infective agents.

**Scheme 49:**
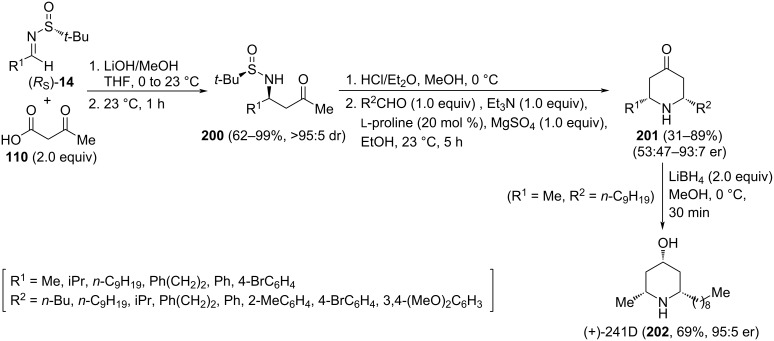
Stereoselective synthesis of *cis*-2,6-disubstituted piperidines **200**, and alkaloid (+)-241D, from chiral imines (*R*_S_)-**14**.

The base-promoted decarboxylative Mannich coupling of chiral imines (*R*_S_)-**14** [derived from an aldehyde R^1^CHO and (*R*)-*tert*-butanesulfinamide] with these reagents provided β-amino ketone derivative **200** in high yields and diastereoselectivities [120]. These compounds were easily transformed into *cis*-2,6-disubstituted piperidin-4-ones **201** through a ʟ-proline organocatalyzed intramolecular Mannich reaction with a second aldehyde (R^2^CHO). Almost no diastereoselectivity was observed when R^1^ and R^2^ were aromatic rings. On the other hand, aliphatic aldehydes gave in general excellent enantiomeric ratios (>90:10). It is important to note that the order of reaction of carbonyl compounds R^1^CHO and R^2^CHO with *tert*-butanesulfinamide to form chiral imine **14**, or in the intramolecular organocatalyzed condensation, determined the absolute configuration of compounds **201** [141]. The usefulness of this methodology was demonstrated in the synthesis of the alkaloid (+)-241D (**202**), isolated from the skin of the Panamanian poison frog *Dendrobates speciosus*, through the reduction of piperidin-4-one **201** (R^1^ = Me, R^2^ = *n*-C_9_H_19_) with lithium borohydride (Scheme 49).

**Scheme 50:**
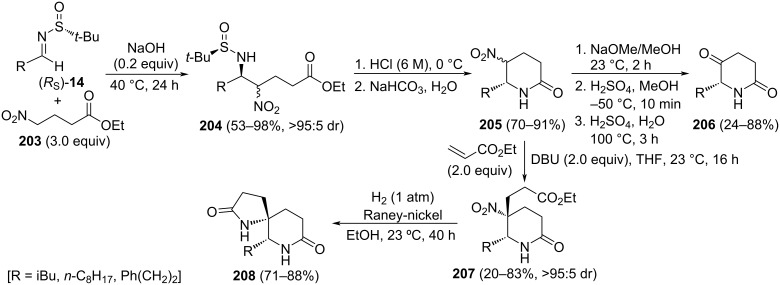
Stereoselective synthesis of 6-substituted piperidines-2,5-diones **206** and 1,7-diazaspiro[4.5]decane-2,8-diones **208** from chiral imines (*R*_S_)-**14**.

The base-catalyzed addition of 4-nitrobutanoates **203** to *N*-*tert*-butanesulfinyl imines (*R*_S_)-**14** under solvent-free reaction conditions proceeded with high facial diastereoselectivity. The resulting β-nitroamine derivatives **204** were easily transformed into 5-nitro-6-substituted piperidine-2-ones **205**, upon removal of the sulfinyl group with concomitant δ-lactam formation. Further transformation of the nitro group under Nef-type reaction conditions led to enantioenriched 6-substituted piperidine-2,5-diones **206** [142]. Interestingly, from compounds **205**, and following a two-step process, involving conjugative addition to ethyl acrylate with formation of **207** as a single diastereoisomer, and final reduction of the nitro group with Raney-nickel, 1,7-diazaspiro[4.5]decane-2,8-diones **208** were accessed in a highly stereoselective fashion (Scheme 50) [143].

**Scheme 51:**
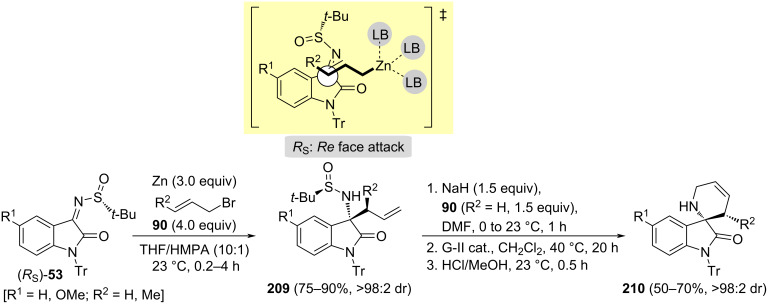
Stereoselective synthesis of spirocyclic oxindoles **210** from chiral imines (*R*_S_)-**53**.

A large number of biologically active natural products and synthetic pharmaceutical drugs contain the 3-aminooxidole motif. Chen and Xu demonstrated that the zinc-mediated allylation of chiral oxindole sulfinyl imines (*R*_S_)-**53** with allylic bromides proceeded smoothly at room temperature in a mixture of THF and HMPA, and a wide range of highly enantiomerically enriched 3-allyl-substituted 3-aminooxindoles **209** were prepared. The observed diastereofacial selectivity was rationalized by considering an acyclic transition state model. The addition of the allylic reagent occurred to the less hindered *Re* face of the imine with (*R*_S_)-configuration. *N*-Allylation of compounds **209**, followed by ring-closing metathesis with Grubbs second generation catalyst, and removal of the sulfinyl group, led to chiral spirocyclic aminooxindoles **210** in reasonable yields (Scheme 51) [144].

**Scheme 52:**
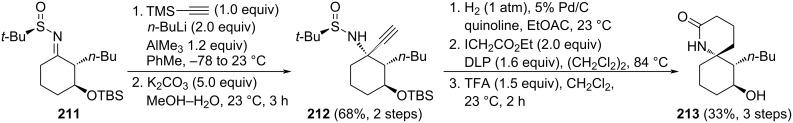
Stereoselective synthesis of azaspiro compound **213** from chiral imine **211**.

Azaspirocyclic alkaloids with interesting pharmacological properties have been isolated from skin extracts of dendrotabic frogs, and also from methanol extracts of ants of the species *Carabella bicolor*. Amongst these alkaloids, (−)-histrionicotoxin displays a potent noncompetitive acetylcholine antagonist activity. It was found that perhydrohistrionicotoxin analogues display similar biological properties. In this regard, Peralta-Hernández and Cordero-Vargas reported the synthesis of an advanced synthetic intermediate **213** of perhydrohistrionicotoxin [145]. The diastereoselective nucleophilic addition of a lithium acetylide to cyclic chiral *N*-*tert*-butanesulfinyl imine **211** is a key step in this strategy. The addition proceeded with total stereocontrol to give a single diastereoisomer, and further removal of the silyl group provided propargylamine derivative **212**. Partial hydrogenation of this compound under Lindlar conditions led to terminal olefin, which reacted with ethyl iodoacetate in refluxing dichoroethane, in the presence of 1.6 equivalents of lauroyl peroxide (DLP), as thermal initiator of the radical process. A spirolactam was isolated in 45% yield, taking place under the essayed reaction conditions successively a radical addition of the enolate to the terminal alkene, lactonization and removal of the sulfinyl group. Final deprotection of the hydroxy group led to compound **213**, a precursor of the 6-(*R*) epimer of perhydrohistrionicotoxin (Scheme 52).

**Scheme 53:**
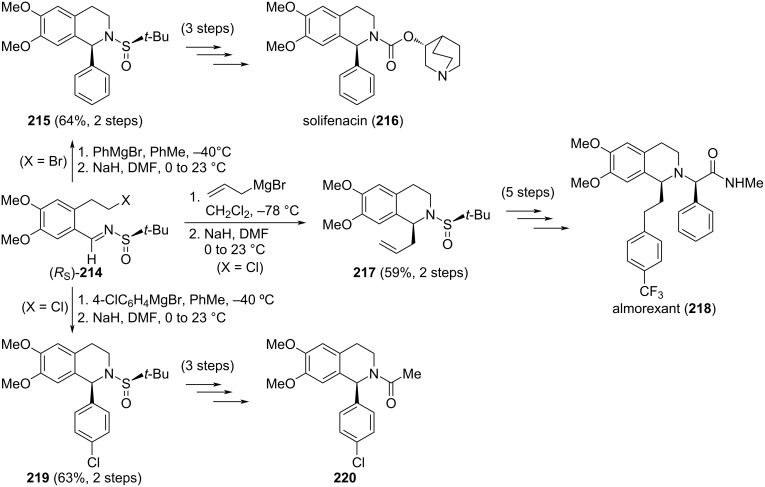
Stereoselective synthesis of tetrahydroisoquinoline derivatives from chiral imines (*R*_S_)-**214**.

Chiral aromatic sulfinyl imines **214** with a 2-haloethyl substituent at *ortho*-position were effective synthetic intermediates in the stereoselective preparation of 1-aryl-1,2,3,4-tetrahydroisoquinolines, which are compounds that display interesting biological activities. For instance, solifenacin (**216**), a competitive muscarinic acetylcholine receptor antagonist currently used in the treatment of overactive bladders, was prepared from (*R*_S_)-**214a** (X = Br) by addition first of phenylmagnesium bromide at –40 °C in toluene. A 93:7 diastereomeric mixture was obtained and the major diastereoisomer was easily isolated after column chromatography. Subsequent intramolecular cyclization in the presence of NaH in DMF at room temperature gave the pure diastereomer **215**, a precursor of solefinacin (**216**) [146]. Diastereoselective allylation of chlorinated derivative (*R*_S_)-**214b** (X = Cl) with allylmagnesium bromide in dichloromethane gave the corresponding homoallylic sulfinamide as a 9:1 mixture of easily separable diastereoisomers, and the major component of the mixture was further cyclized to give product **217**, which was transformed after 5 steps into almorexant (**218**), a non-peptide antagonist of the human orexin receptor, which plays a major role in controlling the sleep/wake cycle [147]. The same precursor (*R*_S_)-**214b** (X = Cl) and strategy was followed in the first steps of the synthesis of compound **220**, used as neuroprotective agents in the treatment of neurological diseases, such as epilepsy and ischemia. In this case, the addition of 4-chlorophenylmagnesium bromide to (*R*_S_)-**214b** gave the expected product with 93:7 ratio of diastereoisomers. An intramolecular cyclization of the major diastereoisomer afforded **219** in high yield (Scheme 53) [146].

### Asymmetric synthesis of pyrrolizidines, indolizidines and quinolizidines

Bicyclic systems containing bridgehead nitrogen, such as 1-azabicyclo[3.3.0]octanes, 1-azabicyclo[4.3.0]nonanes and 1-azabicyclo[4.4.0]decanes are structural motifs frequently encountered in alkaloids, which can come from quite diverse sources, such as bacteria, fungi, plants and animals, among others. Many of these natural products display extremely potent biological activities and their syntheses, along with that of structurally related analogs, remain of great interest for chemists and pharmacologists. Relevant contributions regarding the synthesis of these bicyclic compounds, involving chiral *N*-*tert*-butanesulfinyl imines, are compiled in the following paragraphs.

#### Initial stereocontrol by allylation of sulfinyl imines

The allylation of chiral *N*-*tert*-butanesulfinyl imines is of great synthetic interest because in this reaction together with a new functionality (amino derivative group), a carbon–carbon bond is formed. In addition, the double bond of the allylic moiety can participate in a number of further synthetically useful transformations, including the generation of functional groups prone to participate in intramolecular cyclization processes involving the nitrogen atom of the starting imine. Interestingly, the allylation of these imines can be carried out in a stereoselective fashion with different allylating reagents [66,148–149].

**Scheme 54:**
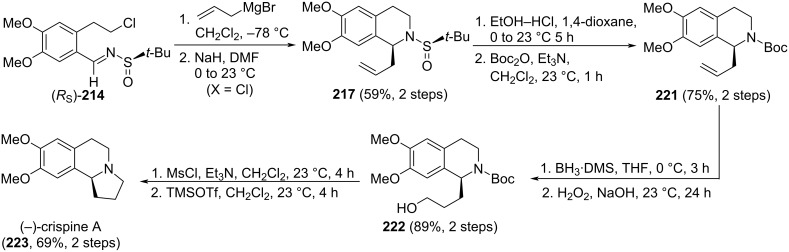
Stereoselective synthesis of (−)-crispine A **223** from chiral imine (*R*_S_)-**214**.

Pyrroloisoquinoline alkaloid (−)-crispine A (**223**) was isolated from *Carduus crispus* plants which were used in folk medicine for the treatment of different inflammatory diseases, such as bronchitis, stenocardia, gastroenteritis, and rheumatism. In addition, it also shows promising biological activity against human cancer cell lines. The allylation of chiral imine (*R*_S_)-**214** with allylmagnesium bromide in dichloromethane at −78 °C was the key step in the synthesis reported by Reddy and co-workers of this alkaloid. The allylated product was obtained in 80% yield as 9:1 mixture of diastereoisomers, and the major diastereoisomer was separated from the mixture and cyclized to give tetrahydroisoquinoline **217** (see above, Scheme 53). The formation of the 5-membered ring to produce target (−)-crispine A (**223**) was accomplished in six additional steps which comprise removal of the sulfinyl group and subsequent *N*-Boc protection to give **221**, hydroboration–oxidation to produce terminal alcohol derivative **222**, and formation of the mesylate, removal of the Boc group, and final cyclization (Scheme 54) [150].

**Scheme 55:**
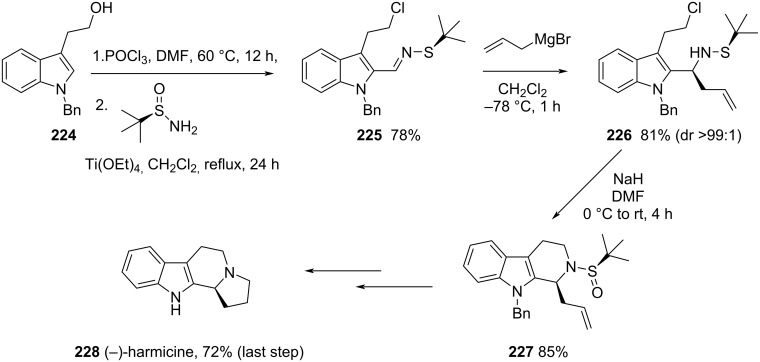
Synthesis of (−)-harmicine (**228**) using *tert*-butanesulfinamide through haloamide cyclization.

A similar strategy was described by the same authors for the stereoselective synthesis of (−)-harmicine and other tetrahydro-β-carboline alkaloids. The allylation of chiral imine (*R*_S_)-**225** with allylmagnesium bromide in dichloromethane at −78 °C was the key step for the synthesis reported by Reddy and co-workers that led to compound **226** in 81% yield (dr > 99:1). After a sequence of similar steps of removal of the sulfinyl group, protection of the amine, hydroboration–oxidation and formation of the mesylate, removal of the Boc group, and final cyclization, the (−)-harmicine (**228**) was obtained in 72% yield in the last step [151] (Scheme 55).

**Scheme 56:**
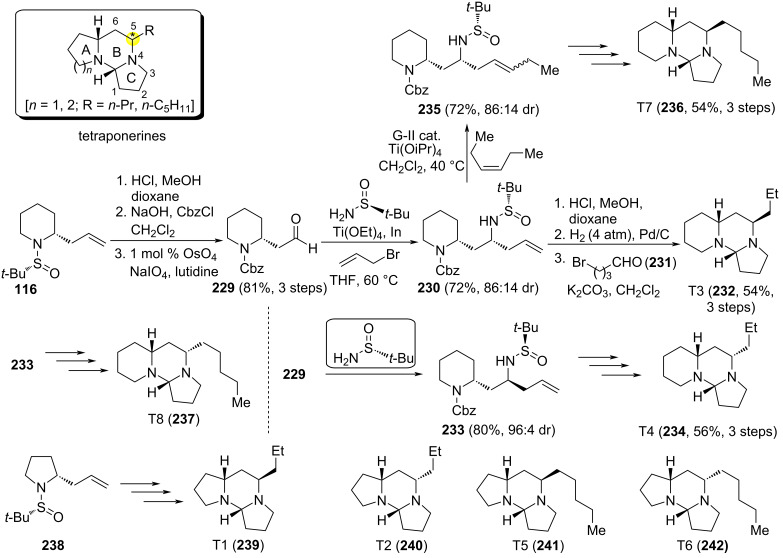
Stereoselective synthesis of tetraponerines T1–T8.

Tetraponerines T1–T8 are tricyclic alkaloids with aminal structure, and depending on the size of the A–B–C rings, they are divided in two groups (5−6−5: T1, T2, T5, T6; or 6−6−5: T3, T4, T7, T8). Other differentiating elements are the alkyl chain at C5-position (*n*-propyl: T1–T4; *n*-pentyl: T5–T8), and the configuration of this stereocenter [(*R*): T1, T3, T5, T7; (*S*): T2, T4, T6, T8] (Scheme 56). The stereocontrolled synthesis of these alkaloids was reported by Bosque et al. Key step transformations in the stereoselective synthesis of each natural tetraponerine are two consecutive indium-mediated aminoallylations of the appropriate stereoisomer of a chiral *N*-*tert*-butylsulfinamide. Allylpyrrolidine derivative **238**, which is the precursor of 5−6−5 tetraponerines (T1, T2, T5, T6; **185**–**187**), was obtained from 4-bromobutanal (**231**) in the first aminoallylation, and allyl piperidine derivative **126** (see Scheme 32; precursor of 6−6−5 tetraponerines: T3, T4, T7, T8; **231**, **233**, **235** and **237**, respectively) was prepared from 5-bromopentanal. Importantly, to prepare tetraponerines T5–T8, with a pentyl group at C5-position, a cross-metathesis reaction involving the allyl group of the second aminoallylation with *cis*-3-hexene was carried out in order to elongate the side chain [152–153]. The anticancer activity of tetraponerines T5–T8 against four different carcinoma human cell lines was also investigated, observing a promising cytotoxic activity of tetraponerine T7 (**236**) against breast carcinoma cell line MCF-7 [152].

**Scheme 57:**
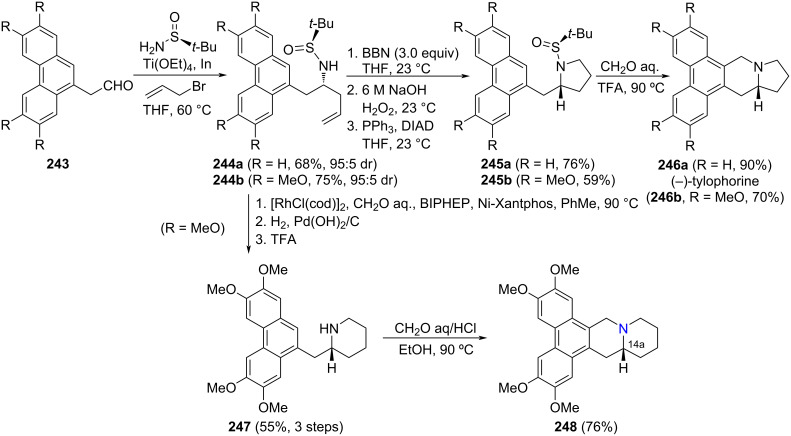
Stereoselective synthesis of phenanthroindolizidines **246a** and (−)-tylophorine (**246b**), and phenanthroquinolizidine **248**.

Taking advantage of this highly diastereoselective indium-mediated amino allylation of carbonyl compounds, an efficient stereocontrolled synthesis of phenanthroindolizidines **246** and phenanthroquinolizidine 7-methoxycryptopleurine **248** was accomplished by Antón-Torrecillas et al., using 2-(phenanthren-9-yl)acetaldehydes **243** as starting materials. The initially formed homoallylamine derivatives **244** were transformed first into pyrrolidines **245** (hydrobroration–oxydation–intramolecular Mitsunobu *N*-alkylation), and after removal of the sulfinyl group, and a Pictet–Spengler reaction involving formaldehyde, the expected phenanthroindolizidine **246a** and the alkaloid (−)-tylophorine (**246b**) were obtained (Scheme 57) [154]. On the other hand, key chiral homoallyllic sulfinamine intermediate **244b** was also transformed in four steps into enantioenriched 7-methoxycryptopleurine **248**, a rhodium-catalyzed linear hydroformylation being one of the steps involved in the formation of piperidine derivative **247**. Cytotoxic evaluation of both enantiomers of 7-methoxycryptopleurine demonstrated that the compound with (*R*)-configuration shown in Scheme 57 was much more potent than its antipode against four cancer cell lines examined [155]. Phenanthroquinolizidines with a quaternary center at C-14a position, bearing a methyl group instead of the proton, were prepared following the same methodology, and using the corresponding methyl ketone as starting material. These compounds displayed also cytotoxic activity against different human cancer cell lines [156].

**Scheme 58:**
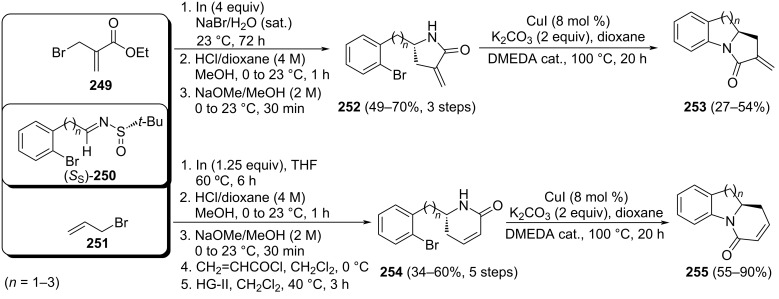
Stereoselective synthesis of indoline, tetrahydroquinoline and tetrahydrobenzazepine derivatives **253** and **255** from chiral aldimines (*S*_S_)-**250**.

Indium-mediated allylation of sulfinyl imines was also the source of the stereocontrol in the synthesis of benzo-fused 1-azabicyclo[*j*.*k*.0]alkanes **253** and **255** reported by Sirvent et al.. The starting chiral imines (*S*_S_)-**250** derived from aliphatic aldehydes, with a 2-bromophenyl substituent, and (*S*)-*tert*-butanesulfinamide. When the allylation was carried out with ethyl 2-(bromomethyl)acrylate (**249**), and after removal of the sulfinyl unit, the resulting free amine derivative led to α-methylene-γ-butyrolactams **252**. On the other hand, dihydropyridin-2-ones **254** were obtained after sequential allylation with allyl bromide **251**, desulfinylation, acylation with acryloyl chloride, and ring-closing metathesis. Lactams **252** and **254** were easily transformed into target polycyclic compounds **253** and **255** by performing an intramolecular *N*-arylation using Ullmann-type reaction conditions (Scheme 58) [157].

**Scheme 59:**
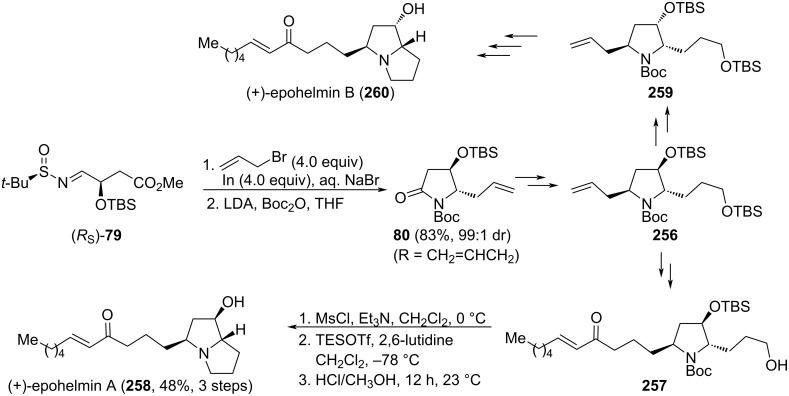
Stereoselective synthesis of (+)-epohelmin A (**258**) and (+)-epohelmin B (**260**) from aldimine (*R*_S_)-**79**.

The group of Wei reported a diastereoselective approach for the synthesis of *trans*-4-hydroxy-5-allyl-2-pyrrolidinone **80** through an indium-mediated allylation of α-chiral aldimine (*R*_S_)-**79** (see above Scheme 24) [100]. Allyl pyrrolidone **80** was an intermediate in the synthesis of alkaloids (+)-epohelmins A (**258**) and B (**260**). These natural products were isolated from an unidentified fungus, and inhibited recombinant lanosterol synthase with low IC_50_ values, with potential use as anticholesteraemic drugs to complement or even substitute the now widely used members of the statin family. The second five-membered ring of the pyrrolizidinic system in (+)-epohelmin A (**258**) was constructed from alcohol **257**, upon forming the corresponding mesylate, subsequent removal of the Boc group, promoting cyclization, and final desilylation. Compound **256** was the last common intermediate in the diastereodivergent approach to both epohelmins, the corresponding epimer **259** was the precursor of (+)-epohelmin B (**260**) by applying the same reaction conditions as for (+)-epohelmin A (**258**, Scheme 59) [158].

**Scheme 60:**
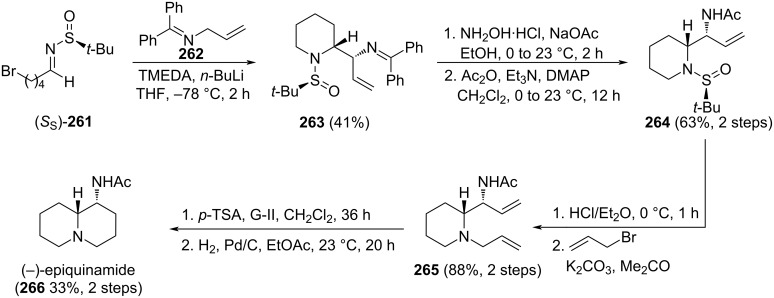
Stereoselective synthesis of (−)-epiquinamide (**266**) from chiral aldimine (*S*_S_)-**261**.

The addition of a lithium anion of *N*-(diphenylmethylidene)allylimine (**262**) to chiral sulfinyl imines was investigated by Prasad and co-workers. They found that the reaction with imines derived from aliphatic aldehydes afforded 1,2-diamine derivatives with excellent diastereoselectivity (>99:1). Allylation of chiral imine (*S*_S_)-**261** with concomitant cyclization led to piperidine **263** as a single diastereoisomer. Removal of diphenylmethylidene group and acylation of the resulting free amine provided compound **264**, which after desulfinylation, *N*-allylation, ring-closing metathesis and catalytic hydrogenation produced the quinolizidine alkaloid (−)-epiquinamide (**266**), isolated from the skin of the Ecuadoran frog *Epipedobates tricolor* (Scheme 60) [159].

**Scheme 61:**
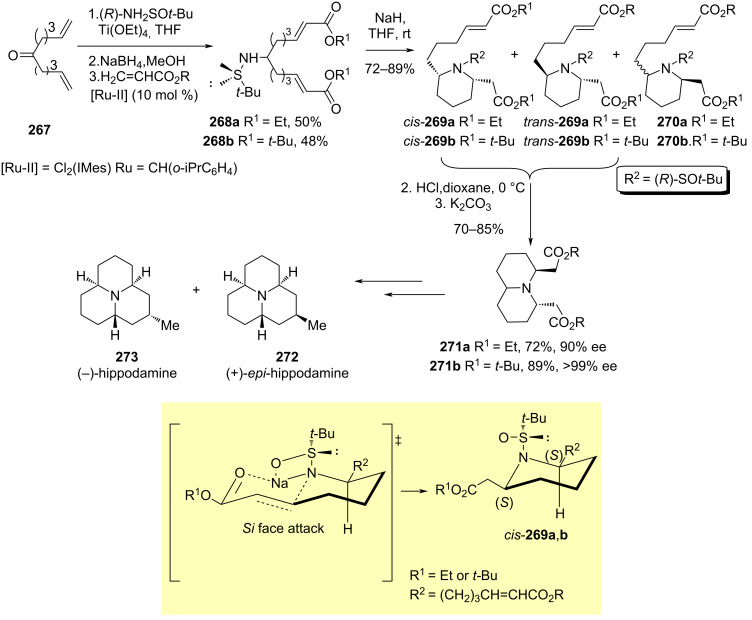
Synthesis synthesis of (–)-hippodamine (**273**) and (+)-*epi*-hippodamine (**272**) using chiral sulfinyl amines.

Fustero and co-workers described for the first time the use of *N*-*tert*-butanesulfinamide in a desymmetrization-type process involving an intramolecular aza-Michael reaction for obtaining the advanced intermediates **271a** and **271b** in the total synthesis of (−)-hippodamine (**273**) and (+)-*epi*-hippodamine (**272**). The condensation reaction between the symmetric ketone **267** and (R)-*N*-*tert*-butanesulfinamide in the presence of titanium(IV) ethoxide followed by the reductive amination with NaBH_4_ and double-direction cross-metathesis reaction led to **268a** in 50% yield and **268b** in 49% yield. These compounds were submitted to the desymmetrization process by an intramolecular aza-Michael reaction using NaH in THF. The applied conditions yielded a mixture of *cis*-**269** and *trans*-**269** diastereoisomers as major product (*cis*/*trans* 3:1) and a small amount of other possible isomer **270** was detected (**269a**/**270a** 95:5) and (**269b**/**270b** 96:4). The addition of nitrogen nucleophile occurred to the *Si* face of the conjugated ester, opposite to the bulky *tert*-butyl group gave *cis-***269a**,**b** as major isomer (Scheme 61). The sulfoxide auxiliary was removed under acid conditions, and after a basification process with saturated aqueous NaHCO_3_, a second intramolecular aza-Michael reaction took place to the products **271a** and **271b** with excellent yields and high diastereoselectivity. Since **271a**,**b** is *C*_2_-symmetric, the cyclization of *cis-***269a**,**b** or *trans*** 269a**,**b** as soon as **270a**,**b** gave the same isomer **271**. Consequently, **271a** was obtained in 90% ee and **271b** in >99% ee, as determined by chiral HPLC analysis. After three steps, a mixture of (−)-hippodamine (**273**) and (+)-*epi*-hippodamine (**272**) were obtained in high yield which was separated by column chromatography [160] (Scheme 61).

### Stereocontrol synthesis through addition of Grignard reagents to *N*-sulfinyl imines

**Scheme 62:**
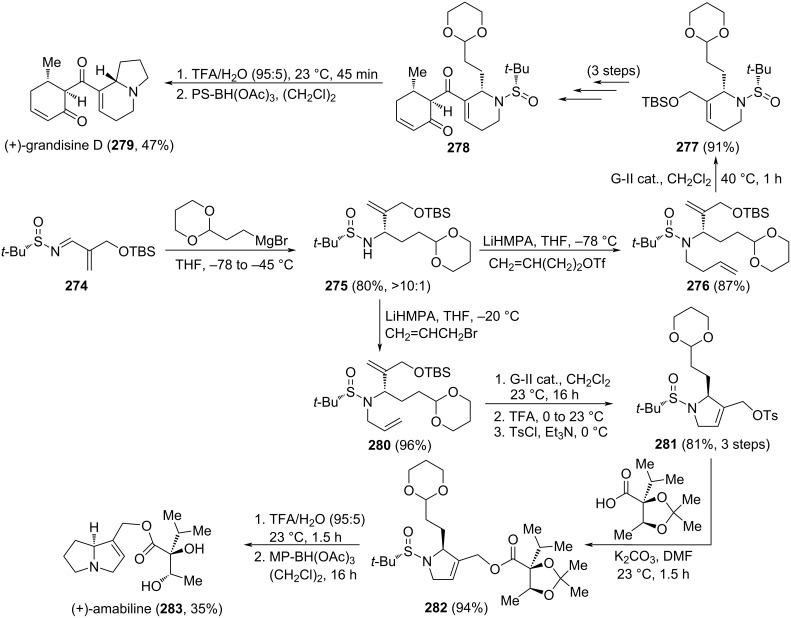
Stereoselective synthesis of (+)-grandisine D (**279**) and (+)-amabiline (**283**).

The group of Lindsley developed new methodologies for the enantioselective synthesis of different azabicyclic systems. These methodologies are based on the diastereoselective addition of Grignard reagents to chiral sulfinyl imines, and applied to the syntheses of the indolizidine alkaloid (+)-grandisine D (**279**) [161], and pyrrolizidine alkaloid (+)-amabiline (**283**) [162], starting from imine **274**. The addition of a masked acetal organomagnesium compound to sulfinyl imine **274** provided compound **275** in high yield and diastereoselectivity, which after reaction with the appropriate alkylating reagent led to **276** and **280**. These compounds were prone to undergo ring-closing metathesis, generating 6- and 5-membered ring systems, respectively. After several steps, acetal derivatives **278** and **282** were transformed into (+)-grandisine D (**279**) and (+)-amabiline (**283**). Treatment under acidic conditions of **278** and **282** produced removal of the sulfinyl group and hydrolysis of the acetal, revealing the masked aldehyde. In the final step, an intramolecular hydroamination allowed the formation of the five-membered ring, to complete the construction of the azabicyclic arrays (Scheme 62). The indolozidine alkaloids grandisines were isolated from leaves of the Australian rain forest tree *Elaeocarpus grandis*, and these alkaloids display selective human δ-opioid receptor affinity. Meanwhile, hepatotoxic (+)-amabiline (**283**) was found in the seeds and flowers of *Borago officinalis*.

**Scheme 63:**
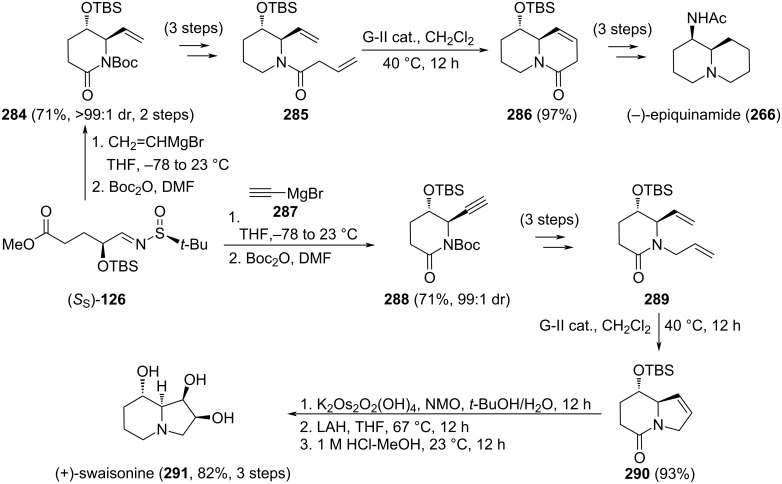
Stereoselective synthesis of (−)-epiquinamide (**266**) and (+)-swaisonine (**291**) from aldimine (*S*_S_)-**126**.

The addition of alkynyl or alkenyl Grignard reagents to chiral sulfinyl imine (*S*_S_)-**126** led to 6-substituted *trans*-5-hydroxy-2-piperidinones with high diastereoselectivity, which is controlled mainly for the configuration of the stereocenter bearing the silyloxy group with alkenyl reagents, and by the coordination of the silyloxy substitution and stereochemistry of the sulfinamide with alkynyl organomagnesium compounds. The addition of vinylmagnesium bromide to (*S*_S_)-**126** led, after *N*-Boc protection to 2-piperidinone **284**, a precursor of diolefin **285**, which through ring closing metathesis allowed the synthesis of quinolizidinone **286**, from which (−)-epiquinamide (**266**) was prepared after three steps. On the other hand, the reaction of imine (*S*_S_)-**126** with ethynylmagnesium bromide provided compound **288**, which was transformed into the *N*-allyl-substituted piperidinone **289**. Again, a ring-closing metathesis allowed the formation of the bicyclic indolizidinone system **290**, an advanced synthetic intermediate of alkaloid (+)-swainsonine (**291**, Scheme 63) [163]. This alkaloid was found in the fungus *Rhizoctonia leguminicola* and in plants such as the Australian *Swainsona canescens* and the North American locoweed *Astragalus lentiginosus*. It displays a wide range of biological activities, including alteri neoplastic growth and metastasis.

### Stereocontrol synthesis through Mannich, nitro-Mannich and Mukaiyama–Mannich reactions of *N*-sulfinyl imines

**Scheme 64:**
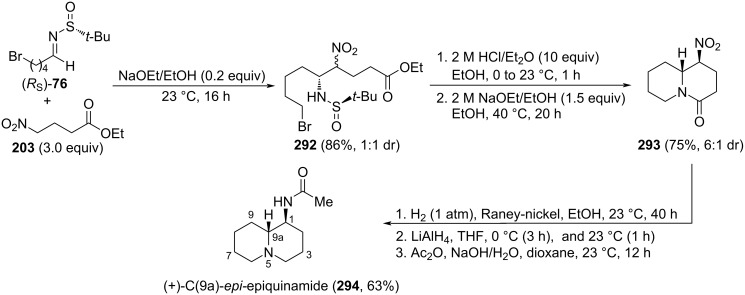
Stereoselective synthesis of (+)-C(9a)-*epi*-epiquinamide (**294**).

Heterocyclic compounds can be obtained through strategies using Mannich reactions [164–166]. It was previously commented that the addition of ethyl 4-nitrobutanoante (**203)** to chiral sulfinyl imines under basic solvent-free conditions proceeded with high facial diastereoselectivity (Scheme 64 [143]). When the reaction was performed with the imine of 5-bromopentanal, (*R*_S_)-**76**, the nitro-Mannich adduct **292** was formed in 86% yield as a 1:1 mixture of diastereoisomers, considering the stereocenter bearing the nitro group. Taking advantage of this methodology, Benlahrech et al. reported the synthesis of (+)-C(9a)-*epi-*epiquinamide (**294**) [167]. Desulfinylation of compound **292** led to quinolizidine derivative **293**, taking place two consecutive cyclizations involving the resulting free amino group, the ester and the alkyl bromide functionalities. Although compound **292** was isolated in a 1:1 dr, quinolizidine **293** was formed as a 6:1 mixture of diastereoisomers, due to a rapid epimerization under basic conditions, leading to the thermodynamic most stable isomer. Sequential reductions of the nitro group, the lactam and final *N*-acetylation of the primary amine were the last steps of this synthesis (Scheme 64).

**Scheme 65:**
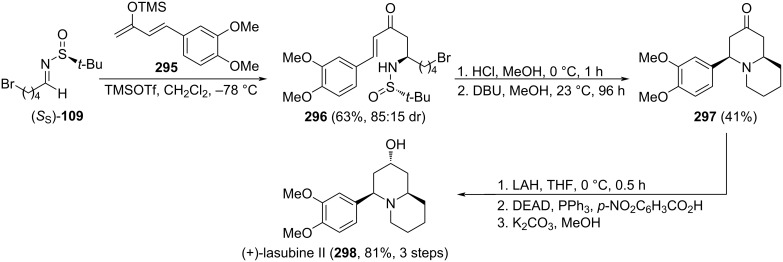
Stereoselective synthesis of (+)-lasubine II (**298**) from chiral aldimine (*S*_S_)-**109**.

The Lewis acid-promoted addition of silyl enol ethers to chiral sulfinyl imines afforded β-amino ketones in high yields and diastereoselectivities (see above Scheme 24 [100]). Similar levels of stereoselectivity were found performing this type of Mukaiyama–Mannich reactions with enol ethers derived from methyl-conjugated enones. The reaction of chiral imine (*S*_S_)-**109** with silyl enol ether **295** led to β-amino enone **296**. Removal of the sulfinyl group under acidic conditions and subsequent basic treatment with DBU led, after *N*-alkylation and intramolecular conjugate addition, to *cis*-quinolizidinone **297** in 41% yield (35% of the *trans*-isomer). Reduction of the ketone functionality with LAH gave 2-*epi-*lasubine II, and Mitsunobu inversion of the configuration provide (+)-lasubine II (**298**) in 81% yield (Scheme 65) [168].

**Scheme 66:**
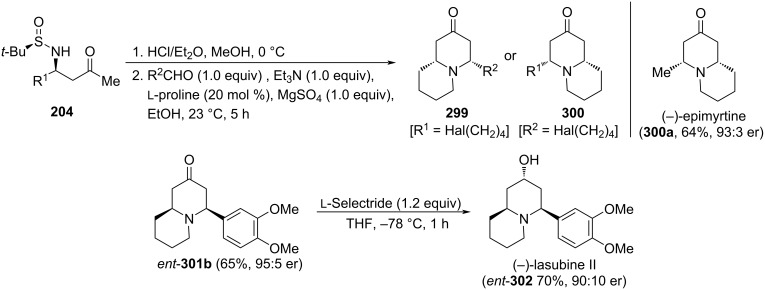
Stereoselective synthesis of (−)-epimyrtine (**300a**) and (−)-lasubine II (*ent*-**302**) from β-amino ketone derivatives **204**.

A straightforward synthesis of the alkaloids (−)-epimyrtine (**300a**) and (−)-lasubine II (*ent*-**302**) from β-amino ketone derivatives **204** was reported by Lahosa et al. These compounds participate in a ʟ-proline organocatalyzed intramolecular Mannich reaction with a second aldehyde (R^2^CHO) to give *cis*-2,6-disubstituted piperidin-4-ones **205** (see above Scheme 49 [141]). However, when one of the aldehydes involved in the formation of β-amino ketone **204** (R^1^CHO) or in the intramolecular Mannich reaction (R^2^CHO) which is 5-chloro- or 5-bromopentanal, quinolizidinone derivatives **299** or **300** are formed. In both cases, after formation of the piperidine ring through the expected Mannich condensation, a subsequent intramolecular *N*-alkylation involving the carbon–halogen bond occurred to produce the quinolizidinic systems. The natural alkaloid (−)-epimyrtine (**300a**), isolated from bilberry (*Vaccinium myrtillus*) was prepared following this methodology, along with *ent*-**299b**, which was transformed in a single step into (−)-lasubine II (*ent*-**302**), by diastereoselective reduction with ʟ-selectride (Scheme 66) [141].

**Scheme 67:**
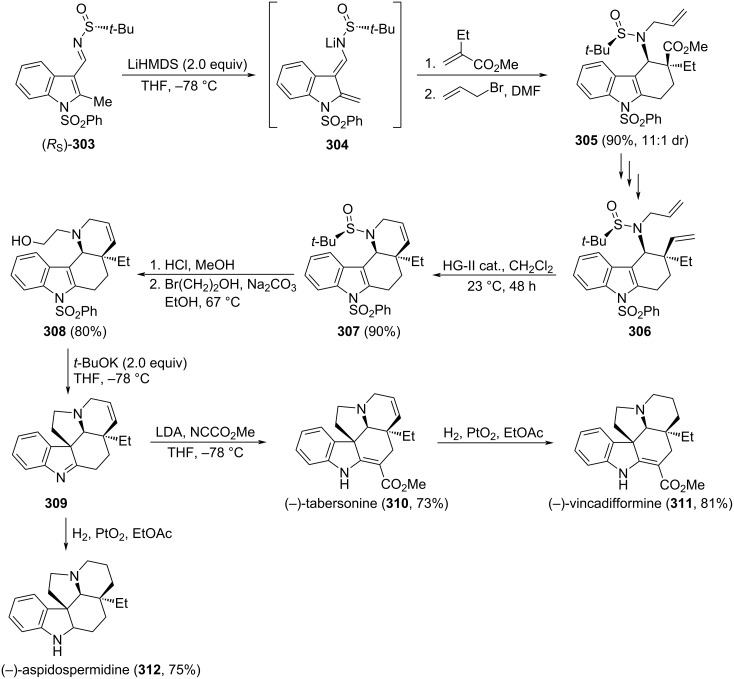
Stereoselective synthesis of (−)-tabersonine (**310**), (−)-vincadifformine (**311**), and (−)-aspidospermidine (**312**).

Andrade [8,169–170] and Zhao reported an elegant and efficient methodology for the rapid assembling of chiral *N*-*tert*-butanesulfinyl imine (*R*_S_)-**303** with methyl 2-ethylacrylate to give tricyclic derivative **305**, in high yield and diastereoselectivity. Treatment of (*R*_S_)-**303** with LiHMDS in THF at –78 °C led to the formation of lithiodienamine intermediate **304**, which reacted with methyl 2-ethylacrylate, leading to the Michael addition product, and the resulting enolate participated in an intramolecular Mannich reaction to form a six-membered ring. Final addition of an excess of allyl bromide yielded tetrahydrocarbazole **305** in 90% yield. After convenient functional group transformation, diolefin **306** was submitted to ring-closing metathesis to form tetracycle **307**. Further removal of the sulfinyl group and *N*-alkylation with 2-bromoethanol gave compound **308**, which is an ideal substrate for the construction of the five-membered ring leading to spiro compound **309**, upon reaction with potassium *tert*-butoxide (Bosch−Rubiralta spirocyclization). Treatment of **309** with LDA at –78 °C and quenching the resulting metalloenamine with methyl cyanoformate furnished (−)-tabersonine (**310**), and selective hydrogenation of **310** led to (−)-vincadifformine (**311**). In addition, double hydrogenation of **309** produced (−)-aspidospermidine (**312**) in 75% yield. All these alkaloids are members of the *Aspidosperma* family (Scheme 67) [171–172].

### Stereocontrol by pinacol-type coupling reactions involving N-sulfinyl imines

**Scheme 68:**
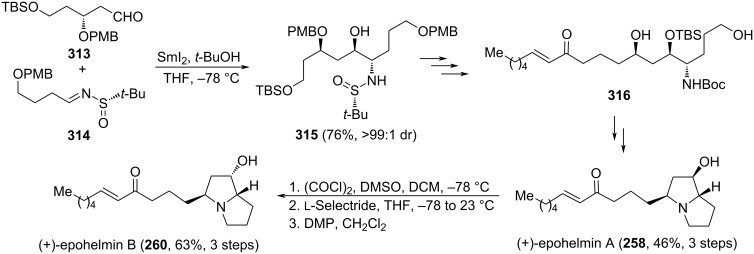
Stereoselective synthesis of (+)-epohelmin A (**258**) and (+)-epohelmin B (**260**) from aldehyde **313** and aldimine **314**.

The alkaloids (+)-epohelmin A (**258**) and (+)-epohelmin B (**260**) were prepared from chiral aldehyde **313** and chiral sulfinyl imine **314**, through samarium iodide-induced reductive cross-coupling reaction leading to compound **315** in 76% yield, and total diastereoselectivity. After several steps, enone amino triol derivative **316** was prepared and directly transformed into (+)-epohelmin A (**258**), by applying the same reaction conditions shown in Scheme 59 [158] for the transformation of pyrrolidine derivative **257** into (+)-epohelmin A (**258**). The epimer (+)-epohelmin B (**260**) was also prepared from **258**, carrying out an oxidation of the alcohol and a diastereoselective reduction with ʟ-selectride of the resulting cyclic ketone (Scheme 68) [173].

### Asymmetric synthesis of lysergic acid

**Scheme 69:**
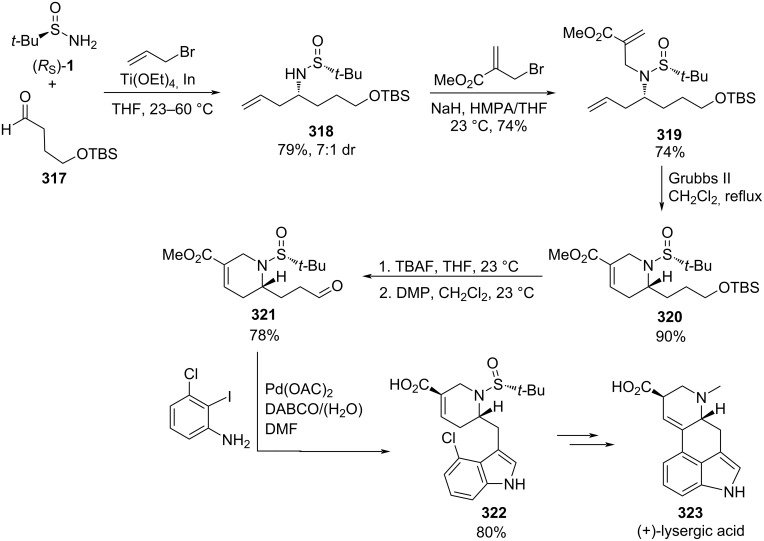
Total synthesis of (+)-lysergic acid (**323**) from *N*-*tert*-butanesulfinamide (*RS*)-**1**.

In 2013, Liu and colleagues used *N*-*tert-*butanesulfinamide in the total synthesis of (+)-lysergic acid (**323**). The first step was the aminoallylation of aldehyde **317** with allyl bromide and sulfinamide (*R*_S_)-**1** by means of indium metal, leading to homoallyl derivative **318**. Further steps included an *N*-alkylation and ring-closing metathesis with Grubbs second generation catalyst to generate tetrahydropyridine **320** in high yields. Removal of the silyl protecting group, followed by oxidation of the resulting terminal alcohol and palladium-catalyzed coupling of the aldehyde with 3-chloro-2-iodoaniline afforded indole derivative **322**, which is an advanced precursor of target (+)-lysergic acid (**323**, Scheme 69) [174].

## Conclusion

Important progress has been made in the development of methodologies that make use of stable *N*-*tert*-butanesulfinyl imines as chiral reagents in diverse synthetic transformations, being possible to recover the chiral auxiliary at the end of the reaction. The great value of enantiomerically enriched amines as synthetic intermediates in the route to complex organic molecules, including natural products (mostly, alkaloids), has boosted the development of nucleophilic addition protocols to these Davis–Ellman *N*-sulfinyl imines, since the pioneering work of Davis`s group and subsequent work of Ellman`s group. Interestingly, the existing working models facilitate the prediction of the major diastereoisomer in most cases, making this technology attractive with the aim of synthetic applications.
